# Chinese Herbal Medicine Combined With First-Generation EGFR-TKIs in Treatment of Advanced Non-Small Cell Lung Cancer With EGFR Sensitizing Mutation: A Systematic Review and Meta-Analysis

**DOI:** 10.3389/fphar.2021.698371

**Published:** 2021-08-27

**Authors:** Yan Lu, Chenbing Sun, Lijing Jiao, Yu Liu, Yabin Gong, Ling Xu

**Affiliations:** ^1^ Department of Oncology, Yueyang Hospital of Integrated Traditional Chinese and Western Medicine, Shanghai University of Traditional Chinese Medicine, Shanghai, China; ^2^ Cancer Disease Project Team of Chinese Evidence-Based Medicine Center of Chinese Medicine, Beijing, China

**Keywords:** chinese herbal medicine, non-small cell lung cancer (NSCLC), epidermal growth factor receptor tyrosine kinase inhibitors (EGFR TKIs), meta-analysis, randomized controlled trials (RCT)

## Abstract

**Background:** Non-small cell lung cancer (NSCLC) is the leading cause of cancer-related deaths worldwide. First-generation epidermal growth factor receptor (EGFR) tyrosine kinase inhibitors (EGFR-TKIs) significantly improve prognosis of advanced NSCLC patients harboring EGFR sensitizing mutation. However, acquired resistance to EGFR-TKIs limits the good outcomes. Chinese herbal medicine (CHM) has been used for NSCLC patients receiving EGFR-TKIs for more than 10°years as an adjuvant treatment.

**Methods:** Studies were searched from China BioMedical Literature, Chinese National Knowledge Infrastructure, Cqvip Database, Wanfang Database, MEDLINE (PubMed), EMBASE (Ovid), Google Scholar, and Cochrane Library from inception to March, 2021. Randomized controlled clinical trials (RCT) comparing EGFR-TKIs + CHM (TKIs + CHM) versus EGFR-TKIs with/without placebo (TKIs ± placebo) in participants with advanced NSCLC harboring EGFR sensitizing mutation were included in this study. Two authors screened all references, assessed the risk of bias and extracted data independently. Data were summarized using hazard ratio (HR) and risk ratios (RR), with 95% confidence intervals (CI) for binary outcomes. Meta-analysis was performed using random effects model. Overall quality of evidence was assessed using GRADE.

**Results:** A total of 9 RCTs (1137 participants, 581 in the TKIs + CHM group and 556 in the TKIs ± placebo group) were included in this review. Only first-generation EGFR-TKIs were included. Most trials included used oral CHM preparations to tonify Qi and/or Yin. Treatment lasted from enrollment until disease progression (PD) or intolerable adverse events (AE). Combination of CHM with EGFR-TKIs improved median progression-free survival (mPFS) (HR,0.59; 95% CI, 0.52–0.68; *P* < 0.00001) and objective response rate (ORR) (RR, 1.23; 95% CI, 1.13–1.34; *P* < 0.00001) compared with used of EGFR-TKIs ± placebo. CHM reduced AE associated with EGFR-TKIs such as cutaneous toxicity (RR, 0.58; 95% CI, 0.46–0.73; *P* < 0.00001) and diarrhea (RR, 0.43; 95% CI, 0.30–0.60; *P* < 0.00001).

**Conclusion:** Combination therapy of CHM and EGFR-TKIs significantly delays acquired resistance while improving ORR to EGFR-TKIs. Furthermore, CHM reduces AE induced by EGFR-TKIs. More international multi-centered, double-blinded, placebo-controlled, well-designed clinical trials are needed in future research.

## Introduction

Non-small cell lung cancer (NSCLC) is the leading cause of cancer-related deaths worldwide (Siegel et al., 2020). The overall 5-years survival rate for NSCLC between 2009 and 2015 in the United States was 25% (Siegel et al., 2020). Most of NSCLC cases are diagnosed at inoperable advanced stage. Discovery of driving genes such as EGFR mutation (mainly exon 19 Del and exon 21L858R), and development of first-generation epidermal growth factor receptor (EGFR) tyrosine kinase inhibitors (EGFR-TKIs), such as gefitinib (Mok et al., 2009; Fukuoka et al., 2011), erlotinib (Zhou et al., 2011; Rosell et al., 2012), and icotinib (Shi et al., 2013; Shi et al., 2017), have significantly improved prognosis of advanced NSCLC patients harboring EGFR sensitizing mutation. Despite initial therapeutic responses, most patients acquire resistance to EGFR-TKIs and report significant disease progress within 9–11°months (Mok et al., 2009; Fukuoka et al., 2011; Rosell et al., 2012; Zhou et al., 2011; Shi et al., 2013; Shi et al., 2017). Therefore, alternative treatment approaches including Chinese herbal medicine (CHM) have been explored to improve effect of EGFR-TKIs thus ensuring long-term survival of NSCLC patients.

EGFR-TKIs impair the Yin-Yang body balance, resulting in drug resistance and adverse events (AE). Therefore, complimenting EGFR-TKIs with CHM can help maintain Yin-Yang balance, delay EGFR-TKI resistance and reduce side effect of EGFR-TKIs (Xu et al., 2017; Jiang et al., 2020; Yu et al., 2019). CHM has been used for treatment of Chinese NSCLC patients receiving EGFR-TKIs for more than 10°years, mainly by tonifying Qi and/or Yin. CHM contains several active compounds that interact with target proteins involved in EGFR-TKI resistance (Bing et al., 2018), in addition to enhancing the inhibition effect of EGFR-TKIs in gefitinib-resistant NSCLC cells by activating PI3-K/Akt-mediated suppress of MUC1 expression (Li et al., 2016). CHM has also been reported to increase the ratio of M1/M2 macrophages by inhibiting autophagy, and suppress the proliferation of EGFR-TKI-resistant cancer cells (Xu et al., 2020). Several clinical trials and systematic reviews report that CHM is effective in delaying EGFR-TKI resistance and alleviating AE (.Yang et al., 2018; Jiao et al., 2019;; Zhang et al., 2018; Sui et al., 2020). However, these trials have relatively small sample size and report inconsistent results. In addition, current systematic reviews and meta-analysis report flawed inclusion criteria as they include patients with unknown EGFR status.

The current study sought to assess benefits and negative effects of CHM as a combination therapy with first-generation EGFR-TKIs in advanced NSCLC patients harboring an active EGFR mutation. Effects of CHM on progression-free survival (PFS), objective response rate (ORR), Karnofsky Performance Status (KPS), quality of life (QoL) and AE were explored.

## Methodology

### Systematic Review Protocol

The protocol used in this review was registered in Inplasy (https://inplasy.com/) on November 16, 2020 (Registration NO: INPLASY2020110063, DOI NO: 10.37766/inplasy 2020.11.0063). The review was conducted and reported according to the Preferred Reporting Items for Systematic Reviews and Meta-Analyses (PRISMA) (Page et al., 2021).

### Inclusion Criteria

The inclusion criteria used in this study were as follows:

1) Type of studies: all randomized controlled trials (RCTs) published in English or Chinese before March 19, 2021 were considered, regardless of blinding.
2) Type of Participants: participants with age ≥18°years, pathologically or cytologically confirmed with inoperable stage III-IV NSCLC, harboring sensitive EGFR mutation and with Eastern Cooperative Oncology Group (ECOG) performance status (PS) scores of 0–3 or Karnofsky Performance Status (KPS) scores above 60 without major organ dysfunction were included in the study.
3) Type of Intervention: TKIs + CHM group received EGFR-TKIs + CHM. CHM was either administered orally, externally or intravenously. EGFR-TKIs included but were not limited to gefitinib, erlotinib, and icotinib.
4) Types of controls: TKIs ± placebo group received the same EGFR-TKIs as intervention group, with or without placebo.
5) Types of outcome measures: primary outcomes were PFS, which was measured with the date of videography from a random assignment to the date of objective progression or death by the researcher. Secondary endpoints included a comparison of overall survival (OS), ORR, disease control rate (DCR), 1- and/or 2-years survival rate, KPS, QoL and safety. QoL was evaluated with the Functional Assessment of Cancer Therapy–Lung (FACT-L) questionnaire. Safety was evaluated based on common terminology criteria for adverse events (CTCAE) version 3.0 guidelines.

### Search Strategy

MEDLINE searching strategy is presented in Supplementary Material S1.

### Study Identification and Selection

Articles were searched in China BioMedical Literature (CBM), Chinese National Knowledge Infrastructure (CNKI), Cqvip database, Wanfang database, MEDLINE (PubMed), embase (Ovid), Google Scholar, and Cochrane Library from inception to March 19, 2021. Search terms included “non-small cell lung cancer”, “NSCLC”, “epidermal growth factor receptor”, “EGFR-TKI”, “traditional Chinese medicine”, “Chinese herbal medicine” and “randomized controlled trial”.

Two authors (Y Lu and CB Sun) independently screened the titles and abstracts of articles using NoteExpress. After screening abstracts, full texts of the articles were downloaded and reviewed. In case of disagreement between the two authors, a third author was involved in the discussion to reach a consensus (LJ Jiao).

### Data Extraction

Two independent reviewers (Y Lu and CB Sun) recorded the data into Microsoft Excel 2010 which comprised the following information: 1) basic information of included studies: ID (Author’s initials + year), publication language, publication year, center locations, sample size, intervention measures, control measures and treatment course; 2) patient information: age, gender, smoking status, staging, pathology type, gene mutation type, TCM pattern differentiation, physical status score, and previous treatment; 3) outcome measurements: primary outcomes: PFS; secondary outcomes: OS, ORR, DCR, 1- and/or 2-years survival rate, QoL, KPS, and toxicity (overall AE, rash, diarrhea, hepatic dysfunction and dental ulcer). 4) Subgroup data based on sex (male *vs* female), age (<65*vs*≥65), ECOG PS (0 *vs* 1 vs 2), staging (IIIa *vs* IIIb *vs* IV), smoking status (yes *vs* no), EGFR mutation status (19 Del *vs* 21L858R *vs* other rare mutations), TKIs (gefitinib *vs* erlotinib *vs* icotinib) and CHM prescriptions (tonifying Qi *vs* tonifying Qi and Yin). Any discrepancies were discussed and where necessary, a third reviewer (LJ Jiao) was involved.

### Quality Assessment

Cochrane Risk Bias Assessment Tool 5.1.0 was used to evaluate the design and methods of included papers, and risk of bias and applicability. Methodological issues related to quality of RCT were generation of treatment allocation, concealment of treatment, blinding, completeness of the resulting data, selective reporting of findings, and other potential risks of bias. Presence of potential biases within the studies was reported descriptively.

### Data Analysis

Meta-analysis was performed using Review Manager 5.4 software. Statistical analysis was performed following the statistical guidelines cited in the latest Cochrane Handbook for Systematic Reviews of Interventions (Higgins and Green 2017). Chi-squared test for heterogeneity was performed, and heterogeneity might not be important when I^2^ was 0–40%. Moderate heterogeneity was present when I^2^ was 30–60%. Substantial heterogeneity was present when I^2^ was 50–90%. Considerable heterogeneity was present when I^2^ was 75–100%, in which case, meta-analysis was not performed and the source of heterogeneity was explored. Random-effect model was used for meta-analysis. Effectiveness on PFS was presented with hazard ratio (HR) and 95% confidential interval (CI). Effectiveness on ORR, DCR and AE were presented with risk ratio (RR) and 95% CI. Description analysis was performed when quantitative data could not be pooled. Funnel plots were generated to analyze potential publication bias. GRADE was used to assess overall quality of evidence (Guyatt et al., 2011).

## Results

### Description of Literature

**FIGURE 1:**
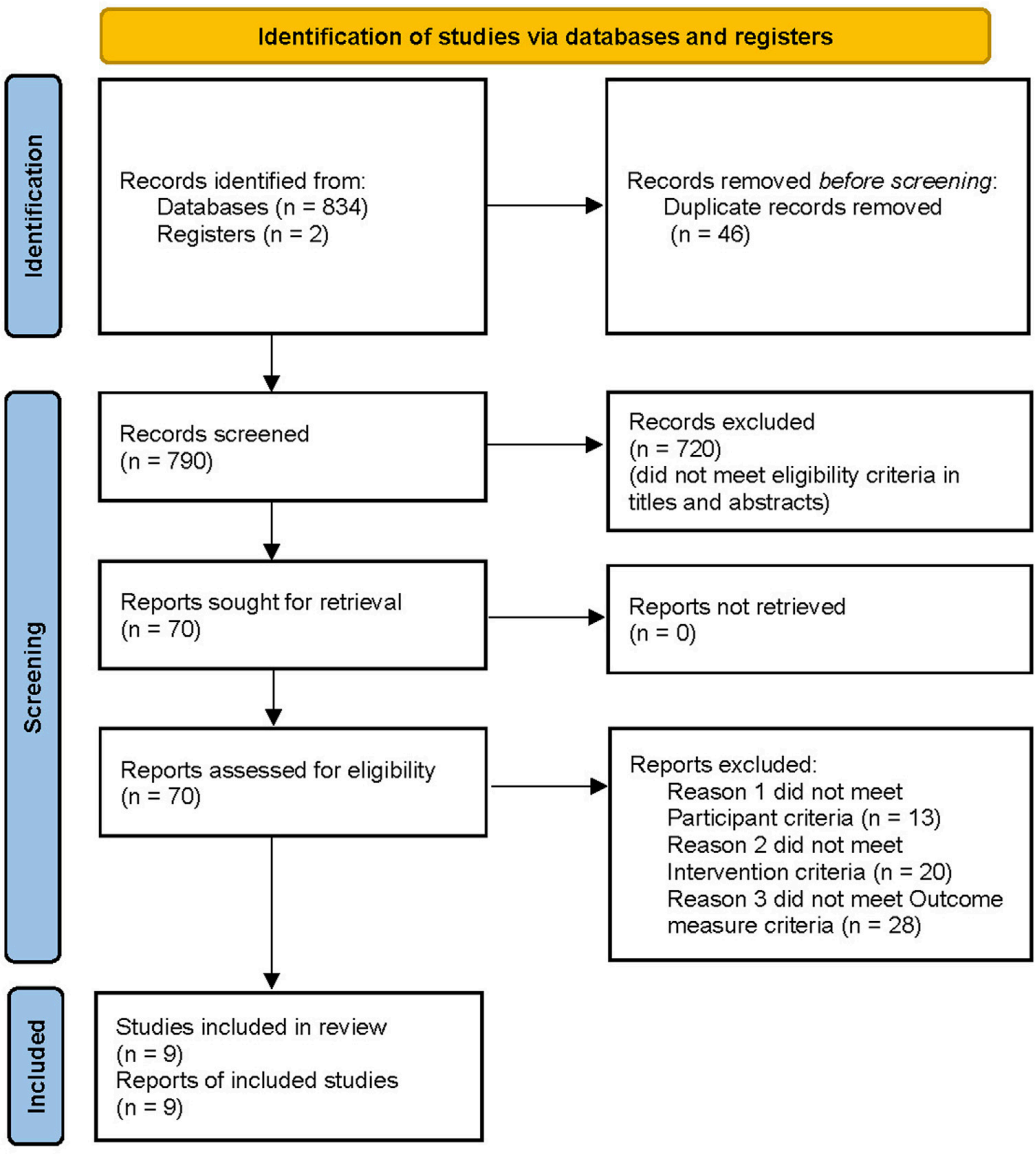
PRISMA flow diagram.

A total of 834 potentially relevant articles were retrieved using the search strategy. Further screening of the 834 studies resulted in exclusion of 46 articles because of duplication. A total of 720 studies were excluded after examining the title and abstract, and 61 studies were excluded after examining the full text, as they did not meet the inclusion criteria. After screening, a total of nine studies were obtained, including two studies were added after a bridging search that was conducted prior to publication of the review. Therefore, the current review included nine studies and a total of 1,137 patients for meta-analysis (Chen 2016; Ju et al., 2017; Yang et al., 2018; Feng et al., 2019; Jiao et al., 2019; Shen et al., 2019; Feng et al., 2020; Zhao et al., 2020; Zhao et al., 2021). The screening process is shown in Figure 1.

### Basic Information of Included Trials

All trials were RCTs, conducted in China from 2009–2018.Three studies were published in English and six in Chinese language. Only the first-generation EGFR-TKIs (gefitinib, erlotinib, and icotinib) were explored in included studies. Gefitinib was the most used drug and was reported in eight studies. All included trials used oral CHM preparations to tonify Qi and/or Yin. Characteristics of studies, participants, disease, and intervention approaches are presented in Table 1. Composition of the preparations are presented in Table 2.

**TABLE 1:** Characteristics of included studies.

First author (publication year)	Rando mization method	Center (n)	Number (n)		Gender n (%)		Age (Mean ± SD, years)	ECOG n (%)		KPS n (%)	Intervention		Outcomes
			T	C	M	F		0–1	2–3		T	C	
Jiao et al. (2019)	CR	15	185	169	130 (36.72)	224 (63.28)	61.0 ± 10.0	338 (95.48)	16 (4.52)	NA	CHM + G/I/E	placebo + G/I/E	PFS, ORR, DCR, QoL, AE
Zhao et al. (2020)	RNT	1	33	31	24 (37.50)	40 (62.50)	<65:36 (56.25); ≥65:28 (43.75)	57 (89.06)	7 (10.94)	NA	CHM + G	Placebo + G	PFS, DCR, QoL, AE
Yang et al. (2018)	RNT	1	35	35	26 (37.14)	44 (62.86)	59.16 ± 11.13	58 (82.86)	12 (17.14)	NA	CHM + G	G	PFS, ORR, DCR, MST, AE
Feng et al. (2020)	RNT	1	65	65	96 (73.85)	34 (26.15)	62.28 ± 9.05	NA	NA	70.95 ± 4.46	CHM + G	G	PFS, ORR, OS, coagulation function, AE
Feng et al. (2019)	NA	1	92	92	103 (55.98)	81 (44.02)	61.03 ± 5.18	131 (71.19)	53 (28.80)	75.47 ± 3.43	CHM + G	G	PFS, ORR, coagulation function, TCM syndrome integral, KPS, AE
Zhao et al. (2021)	RNT	1	58	57	43 (37.39)	72 (62.61)	64.39 ± 10.93	NA	NA	≥70:98 (85.22); <70:17 (14.78)	CHM + G	G	PFS, ORR, DCR, KPS, AE
Shen et al. (2019)	RNT	1	36	35	41 (57.75)	30 (42.25)	71.55 ± 5.38	38 (53.52)	33 (46.48)	NA	CHM + I	I	PFS, Qol, AE
Ju et al. (2017)	RNT	1	45	41	37 (43.02)	49 (56.98)	60.87 ± 10.36	51 (59.30)	35 (40.7)	NA	CHM + G	G	PFS, ORR, DCR, KPS, AE
Chen. (2016)	RNT	2	32	31	23 (36.51)	40 (63.49)	<65:42 (66.67); ≥65:21 (33.33)	NA	NA	NA	CHM + G/I	G/I	PFS, ORR, Qol, TCM syndrome integral, ECOG, AE

**First author (publication year)**	**Histological type n (%)**			**Neoplasm Stage n (%)**		**Gene mutation n (%)**			**Line n (%)**	**TKI n (%)**	**TCM syndrome**	**CHM intervention**
	**A**	**S**	**Other**	**III**	**IV**	**19del**	**21L858R**	**Other**	**1st-2nd-3rd**	**G-E-I**		
Jiao et al. (2019)	354 (100)	0	0	30 (8.49)	324 (91.53)	194 (54.80)	155 (43.79)	5 (1.41)	249 (70.34)-105 (29.66)-0	150 (42.37)-12 (3.39)-192 (54.24)	Yes	Q, Y, QY
Zhao et al. (2020)	61 (95.31)	NA	3 (4.69)	5 (7.81)	59 (92.19)	38 (59.38)	26 (40.62)	0	56 (87.50)-8 (12.50)-0	64 (100.00)-0-0	No	Q
Yang et al. (2018)	65 (92.86)	3 (4.29)	2 (2.86)	5 (7.14)	65 (92.86)	42 (60.00)	28 (40.00)	0	NA	70 (100.00)-0-0	No	Q
Feng et al. (2020)	57 (43.85)	60 (46.15)	13 (10.00)	80 (61.54)	50 (38.46)	NA			NA	130 (100.00)-0-0	Yes	QY
Feng et al. (2019)	NA			143 (77.72)	41 (22.28)	80 (43.48)	104 (56.52)	0	NA	184 (100.00)-0-0	Yes	QY
Zhao et al. (2021)	NA			7 (6.09)	108 (93.91)	NA			NA	115 (100.00)-0-0	Yes	Q
Shen et al. (2019)	NA			4 (5.63)	67 (94.37)	30 (42.25)	36 (50.70)	5 (7.04)	44 (61.97)-25 (35.21)-2 (2.82)	0-0-71 (100.00)	Yes	Q, Y, QY
Ju et al. (2017)	84 (97.67)	1 (1.16)	1 (1.16)	11 (12.79)	75 (87.21)	NA			NA	86 (100.00)-0-0	No	Q, QY
Chen. (2016)	NA			2 (3.18)	61 (96.83)	33 (52.38)	30 (47.62)	0	45 (71.43)-18 (28.57)-0	28 (44.44)-0-35 (55.56)	Yes	Y, QY

ECOG, Zubrod-ECOG-WHO Score; T, treatment group; TKIs + CHM; **C**: control group, TKIs ± placebo; NA: not applicable; M: male; F: female; KPS: Karnofsky Performance Status.

Randomization Method: CR-central randomization; RNT-random number table.

Intervention: CHM-Chinese herbal medicine; G: gefitinib; E: erlotinib; I: icotinib.

Outcome: PFS: progression-free survival; ORR: objective response rate; DCR: disease control rate; KPS: Karnofsky Performance Status; QoL: quality of life; AE: adverse event; MST: median survival time; OS: overall survival.

Histological Type: A-adenocarcinoma; S- squamous carcinoma.

CHM Intervention: Y- tonify Yin; Q: tonify Qi; QY: tonify Qi and Yin.

**TABLE 2:** Composition of the preparations and how these were reported in the original studies.

First author (publication year)	Species, source, concentration	Quality control reported? (Y/N)	Chemical analysis reported? (Y/N)
Jiao et al. (2019)	**Formula I (including tonifying qi and warming yang granules)**	Y - Prepared according to Chinese Pharmacopoeia (2015 edition)	Y – HPLC (Supplementary Figure S5)
	Dried rhizoma of *Astragalus membranaceus (Fisch.)*, [Tianjiang Pharmaceutical Co., Ltd.], 30 g		
	Dried rhizoma of *Codonopsis pilosula (Franch.) Nannf.*, [Tianjiang Pharmaceutical Co., Ltd.], 9 g		
	Dried rhizoma of *Atractylodes macrocephala Koidz.,* [Tianjiang Pharmaceutical Co., Ltd.], 12 g		
	Dried sclerotium of *Poria cocos (Schw.) Wolf*, [Tianjiang Pharmaceutical Co., Ltd.], 15 g		
	Dried plant of *Epimedium sagittatum (Sieb. et Zucc) Maxim.,* [Tianjiang Pharmaceutical Co., Ltd.], 15 g		
	Dried seed of *Trigonella foenum-graecum,* [Tianjiang Pharmaceutical Co., Ltd.], 15 g		
	Fruit of *Psoralea corylifolia L.,* [Tianjiang Pharmaceutical Co., Ltd.], 12 g		
	**Formula II (nourishing yin granules)**		
	Dried root of *Adenophora tetraphylla (Thunb.) Fisch. or Adenophora stricta Miq.,* [Tianjiang Pharmaceutical Co., Ltd.], 30 g		
	Dried rhizoma of *Glehnia littoralis*, [Tianjiang Pharmaceutical Co., Ltd.], 30 g		
	Dried rhizoma of *Radix asparagi,* [Tianjiang Pharmaceutical Co., Ltd.], 15 g		
	Dried rhizoma of *Ophiopogon japonicus*, [Tianjiang Pharmaceutical Co., Ltd.], 15 g		
	Scale leaf of *Lilium brownii*, [Tianjiang Pharmaceutical Co., Ltd.], 15 g		
	Fruit of *Ligustrum lucidum Ait.,* [Tianjiang Pharmaceutical Co., Ltd.], 12 g		
	**Formula III (detoxifying and resolving masses granules)**		
	Dried spikes of *Prunella vulgaris L.*, [Tianjiang Pharmaceutical Co., Ltd.], 7.5 g		
	Dried rhizoma of *Arisaema heterophyllum Blume*, [Tianjiang Pharmaceutical Co., Ltd.], 15 g		
	Dried tubers of *Rhizoma amorphophalli,* [Tianjiang Pharmaceutical Co., Ltd.], 15 g		
	Dried pseudobulb of *Cremastra appendiculate (D.Don) Makino,* [Tianjiang Pharmaceutical Co., Ltd.], 7.5 g		
	Dried plant of *Euphorbia helioscopia*, [Tianjiang Pharmaceutical Co., Ltd.], 7.5 g		
	Dried plant of *Selaginella doederleinii Hieron*, [Tianjiang Pharmaceutical Co., Ltd.], 15 g		
	Dried plant of *Salvia chinensis Benth*, [Tianjiang Pharmaceutical Co., Ltd.], 15 g		
	Dried rhizoma of *Paris polyphylla*, [Tianjiang Pharmaceutical Co., Ltd.], 7.5 g		
	Fruit of *Jujube date,* [Tianjiang Pharmaceutical Co., Ltd.], 4.5 g		
Zhao et al. (2020)	Dried roots of *Pseudostellaria heterophylla (Miq.) Pax ex Pax et Hoffm.*, [Guangdong Yifang Pharmaceutical Co., Ltd.], 30 g	N	N
	Dried rhizoma of *Atractylodes macrocephala Koidz*, [Guangdong Yifang Pharmaceutical Co., Ltd.], 15 g		
	Dried roots of *Astragalus membranaceus (Fisch.)*, [Guangdong Yifang Pharmaceutical Co., Ltd.], 30 g		
	Dried plant of *Oldenlandia diffusa,* [Guangdong Yifang Pharmaceutical Co., Ltd.], 30 g		
	Dried plant of *Solanum nigrum,* [Guangdong Yifang Pharmaceutical Co., Ltd.], 30 g		
	Dried aerial part of *Salvia chinensis Benth.,* [Guangdong Yifang Pharmaceutical Co., Ltd.], 30 g		
	Dried pseudobulb of *Cremastra appendiculate(D.Don) Makino,* [Guangdong Yifang Pharmaceutical Co., Ltd.], 30 g		
	Dried seed kernel of *Coix lacryma-jobi L.var.ma-yuen (Roman.) Stapf,* [Guangdong Yifang Pharmaceutical Co., Ltd.], 30 g		
	Dried fruit of *Akebia quinata (Thunb.) Decne,* [Guangdong Yifang Pharmaceutical Co., Ltd.], 30 g		
	Dried stem and leaf of *Rubus parviflolius L.,* [Guangdong Yifang Pharmaceutical Co., Ltd.], 30 g		
	Dried rhizoma of *Curcumap haeocaulis Val*, [Guangdong Yifang Pharmaceutical Co., Ltd.], 15 g		
	Dried root and rhizoma of *Glycyrrhiza uralensis Fisch.*, [Guangdong Yifang Pharmaceutical Co., Ltd.], 10 g		
Yang et al. (2018)	Dried roots of *Pseudostellaria heterophylla (Miq.) Pax ex Pax et Hoffm.*, [Guangdong Kangmei Pharmaceutical Co., Ltd.], 30 g	Y - Prepared according to CM practice	N
	Dried rhizoma of *Atractylodes macrocephala Koidz*, [Guangdong Kangmei Pharmaceutical Co., Ltd.], 15 g		
	Dried roots of *Astragalus membranaceus (Fisch.)*, [Guangdong Kangmei Pharmaceutical Co., Ltd.], 30 g		
	Dried plant of *Oldenlandia diffusa,* [Guangdong Kangmei Pharmaceutical Co., Ltd.], 30 g		
	Dried plant of *Solanum nigrum,* [Guangdong Kangmei Pharmaceutical Co., Ltd.], 30 g		
	Dried aerial part of *Salvia chinensis Benth.,* [Guangdong Kangmei Pharmaceutical Co., Ltd.], 30 g		
	Dried pseudobulb of *Cremastra appendiculate(D.Don) Makino,* [Guangdong Kangmei Pharmaceutical Co., Ltd.], 30 g		
	Dried seed kernel of *Coix lacryma-jobi L.var.ma-yuen (Roman.) Stapf,* [Guangdong Kangmei Pharmaceutical Co., Ltd.], 30 g		
	Dried fruit of *Akebia quinata (Thunb.) Decne,* [Guangdong Kangmei Pharmaceutical Co., Ltd.], 30 g		
	Dried stem and leaf of *Rubus parviflolius L.,* [Guangdong Kangmei Pharmaceutical Co., Ltd.], 30 g		
	Dried rhizoma of *Curcumap haeocaulis Val*, [Guangdong Kangmei Pharmaceutical Co., Ltd.], 15 g		
	Dried root and rhizoma of *Glycyrrhiza uralensis Fisch.,* [Guangdong Kangmei Pharmaceutical Co., Ltd.], 10 g		
Feng et al. (2020)	Dried root of *Codonopsis pilosula (Franch.) Nannf.*, [Ruikang Hospital affiliated to Guangxi University of Traditional Chinese Medicine], 20 g	N	N
	Dried roots of *Astragalus membranaceus (Fisch.)*, [Ruikang Hospital affiliated to Guangxi University of Traditional Chinese Medicine], 15 g		
	Dried tuberous root of *Rehmannia glutinosa Libosch.,* [Ruikang Hospital affiliated to Guangxi University of Traditional Chinese Medicine], 15 g		
	Dried root and rhizoma of *Aster tataricus L. f.,* [Ruikang Hospital affiliated to Guangxi University of Traditional Chinese Medicine], 10 g		
	Dried rhizomsze of *Ligusticum chuanxiong Hort.,* [Ruikang Hospital affiliated to Guangxi University of Traditional Chinese Medicine], 10 g		
	Dried root of *Paeonia lactiflor Pall.,* [Ruikang Hospital affiliated to Guangxi University of Traditional Chinese Medicine], 12 g		
	Dried root and rhizoma of *Salvia miltiorrhiza Bge.,* [Ruikang Hospital affiliated to Guangxi University of Traditional Chinese Medicine], 10 g		
	Dried root of *Peucedanum praeruptorum Dunn,* [Ruikang Hospital affiliated to Guangxi University of Traditional Chinese Medicine], 10 g		
	Dried seed of *Prunus armeniaca L.* var. *ansu Maxim.,* [Ruikang Hospital affiliated to Guangxi University of Traditional Chinese Medicine], 10 g		
	Dried rhizoma of *Curcumap haeocaulis Val*, [Ruikang Hospital affiliated to Guangxi University of Traditional Chinese Medicine], 10 g		
	Dried rhizoma of *Sparganium stoloniferum Buch. -Ham.,* [Ruikang Hospital affiliated to Guangxi University of Traditional Chinese Medicine], 10 g		
	Dried plant of S*cutellaria barbata D.don,* [Ruikang Hospital affiliated to Guangxi University of Traditional Chinese Medicine], 10 g		
	Dried plant of *Oldenlandia diffusa,* [Ruikang Hospital affiliated to Guangxi University of Traditional Chinese Medicine], 15 g		
	Carapace of *Trionyx sinensis Wiegmann*, [Ruikang Hospital affiliated to Guangxi University of Traditional Chinese Medicine], 10 g		
	Dried root and rhizoma of *Glycyrrhiza uralensis Fisch.,* [Ruikang Hospital affiliated to Guangxi University of Traditional Chinese Medicine], 6 g		
Feng et al. (2019)	Dried root of *Codonopsis pilosula (Franch.) Nannf.*, [Ruikang Hospital affiliated to Guangxi University of Traditional Chinese Medicine], 20 g	N	N
	Dried roots of *Astragalus membranaceus (Fisch.)*, [Ruikang Hospital affiliated to Guangxi University of Traditional Chinese Medicine], 15 g		
	Dried tuberous root of *Rehmannia glutinosa Libosch.,* [Ruikang Hospital affiliated to Guangxi University of Traditional Chinese Medicine], 15 g		
	Dried root and rhizoma of *Aster tataricus L. f.,* [Ruikang Hospital affiliated to Guangxi University of Traditional Chinese Medicine], 10 g		
	Dried rhizomsze of *Ligusticum chuanxiong Hort.,* [Ruikang Hospital affiliated to Guangxi University of Traditional Chinese Medicine], 10 g		
	Dried root of *Paeonia lactiflor Pall.,* [Ruikang Hospital affiliated to Guangxi University of Traditional Chinese Medicine], 12 g		
	Dried root and rhizoma of *Salvia miltiorrhiza Bge.,* [Ruikang Hospital affiliated to Guangxi University of Traditional Chinese Medicine], 10 g		
	Dried root of *Peucedanum praeruptorum Dunn,* [Ruikang Hospital affiliated to Guangxi University of Traditional Chinese Medicine], 10 g		
	Dried seed of *Prunus armeniaca L.* var. *ansu Maxim.,* [Ruikang Hospital affiliated to Guangxi University of Traditional Chinese Medicine], 10 g		
	Dried rhizoma of *Curcumap haeocaulis Val*, [Ruikang Hospital affiliated to Guangxi University of Traditional Chinese Medicine], 10 g		
	Dried rhizoma of *Sparganium stoloniferum Buch. -Ham.,* [Ruikang Hospital affiliated to Guangxi University of Traditional Chinese Medicine], 10 g		
	Dried plant of S*cutellaria barbata D.don,* [Ruikang Hospital affiliated to Guangxi University of Traditional Chinese Medicine], 10 g		
	Dried plant of *Oldenlandia diffusa,* [Ruikang Hospital affiliated to Guangxi University of Traditional Chinese Medicine], 15 g		
	Carapace of Trionyx sinensis Wiegmann, [Ruikang Hospital affiliated to Guangxi University of Traditional Chinese Medicine], 10 g		
	Dried root and rhizoma of *Glycyrrhiza uralensis Fisch.,* [Ruikang Hospital affiliated to Guangxi University of Traditional Chinese Medicine], 6 g		
Zhao et al. (2021)	Dried root and rhizoma of *Panax ginseng C. A. Mey.,* [Zhongshan Hospital of Traditional Chinese Medicine Affiliated to Guangzhou University of Traditional Chinese Medicine], 30 g	N	N
	Dried rhizoma of *Atractylodes macrocephala Koidz*, [Zhongshan Hospital of Traditional Chinese Medicine Affiliated to Guangzhou University of Traditional Chinese Medicine], 15 g		
	Dried sclerotium of *Poria cocos (Schw.) Wolf*, [Zhongshan Hospital of Traditional Chinese Medicine Affiliated to Guangzhou University of Traditional Chinese Medicine], 20 g		
	Dried root and rhizoma of *Glycyrrhiza uralensis Fisch.,* [Zhongshan Hospital of Traditional Chinese Medicine Affiliated to Guangzhou University of Traditional Chinese Medicine], 10 g		
	Dried pericarp of *Citrus reticulate Blanco,* [Zhongshan Hospital of Traditional Chinese Medicine Affiliated to Guangzhou University of Traditional Chinese Medicine], 5 g		
	Dried rhizoma of *Phinellia ternate (Thunb.) Breit.,* [Zhongshan Hospital of Traditional Chinese Medicine Affiliated to Guangzhou University of Traditional Chinese Medicine], 15 g		
Shen et al. (2019)	**Formula I (tonifying yin)**	N	N
	Dried root of *Adenophora tetraphylla (Thunb.) Fisch. or Adenophora stricta Miq.,* [Longhua Hospital Affiliated to Shanghai University of Traditional Chinese Medicine], 15 g		
	Dried root of *Glehnia littoralis Fr. Schmidt ex Miq.,* [Longhua Hospital Affiliated to Shanghai University of Traditional Chinese Medicine], 15 g		
	Dried tuberous root of *Ophiopogon japonicus (Thunb.) Ker. Gawl.,* [Longhua Hospital Affiliated to Shanghai University of Traditional Chinese Medicine], 12 g		
	Dried tuberous root of *Asparagus cochinchinensis(Lour.) Merr.,* [Longhua Hospital Affiliated to Shanghai University of Traditional Chinese Medicine], 12 g		
	Dried fleshy leaf of *Lilium lancifolium Thunb., Lilium brownie F.E.Brown* var. *viridulum Baker or Lilium pumilum DC.,* [Longhua Hospital Affiliated to Shanghai University of Traditional Chinese Medicine], 12 g		
	Dried spikes of *Prunella vulgaris L.*, [Longhua Hospital Affiliated to Shanghai University of Traditional Chinese Medicine], 15 g		
	Shell of *Ostrea gigas Thunberg,* [Longhua Hospital Affiliated to Shanghai University of Traditional Chinese Medicine], 30 g		
	Dried plant of *Selaginella doederleinii Hieron*, [Longhua Hospital Affiliated to Shanghai University of Traditional Chinese Medicine], 30 g		
	Dried plant of *Salvia chinensis Benth.,* [Longhua Hospital Affiliated to Shanghai University of Traditional Chinese Medicine], 30 g		
	Dried plant of *Oldenlandia diffusa,* [Longhua Hospital Affiliated to Shanghai University of Traditional Chinese Medicine], 30 g		
	**Formula II (tonifying qi)**		
	Dried roots of *Astragalus membranaceus (Fisch.)*, [Longhua Hospital Affiliated to Shanghai University of Traditional Chinese Medicine], 15 g		
	Dried root of *Codonopsis pilosula (Franch.) Nannf.*, [Longhua Hospital Affiliated to Shanghai University of Traditional Chinese Medicine], 12 g		
	Dried rhizoma of *Atractylodes macrocephala Koidz*, [Longhua Hospital Affiliated to Shanghai University of Traditional Chinese Medicine], 12 g		
	Dried sclerotium of *Poria cocos (Schw.) Wolf*, [Longhua Hospital Affiliated to Shanghai University of Traditional Chinese Medicine], 9 g		
	Dried plant of *Epimedium sagittatum (Sieb. et Zucc) Maxim.,* [Longhua Hospital Affiliated to Shanghai University of Traditional Chinese Medicine], 15 g		
	Dried spikes of *Prunella vulgaris L.*, [Longhua Hospital Affiliated to Shanghai University of Traditional Chinese Medicine], 15 g		
	Shell of *Ostrea gigas Thunberg,* [Longhua Hospital Affiliated to Shanghai University of Traditional Chinese Medicine], 30 g		
	Dried plant of *Selaginella doederleinii Hieron*, [Longhua Hospital Affiliated to Shanghai University of Traditional Chinese Medicine], 30 g		
	Dried plant of *Salvia chinensis Benth.,* [Longhua Hospital Affiliated to Shanghai University of Traditional Chinese Medicine], 30 g		
	Dried plant of *Oldenlandia diffusa,* [Longhua Hospital Affiliated to Shanghai University of Traditional Chinese Medicine], 30 g		
	**Formula III (tonifying qi and yin)**		
	Dried roots of *Astragalus membranaceus (Fisch.)*, [Longhua Hospital Affiliated to Shanghai University of Traditional Chinese Medicine], 15 g		
	Dried rhizoma of *Atractylodes macrocephala Koidz*, [Longhua Hospital Affiliated to Shanghai University of Traditional Chinese Medicine], 12 g		
	Dried root of *Glehnia littoralis Fr. Schmidt ex Miq.,* [Longhua Hospital Affiliated to Shanghai University of Traditional Chinese Medicine], 15 g		
	Dried tuberous root of *Asparagus cochinchinensis(Lour.) Merr.,* [Longhua Hospital Affiliated to Shanghai University of Traditional Chinese Medicine], 12 g		
	Dried fruit of *Ligustrum lucidum Ait.,* [Longhua Hospital Affiliated to Shanghai University of Traditional Chinese Medicine], 12 g		
	Dried mature fruit of *Lycium barbarum L.*, [Longhua Hospital Affiliated to Shanghai University of Traditional Chinese Medicine], 12 g		
	Dried spikes of *Prunella vulgaris L.*, [Longhua Hospital Affiliated to Shanghai University of Traditional Chinese Medicine], 15 g		
	Shell of *Ostrea gigas Thunberg,* [Longhua Hospital Affiliated to Shanghai University of Traditional Chinese Medicine], 30 g		
	Dried plant of *Selaginella doederleinii Hieron*, [Longhua Hospital Affiliated to Shanghai University of Traditional Chinese Medicine], 30 g		
	Dried plant of *Salvia chinensis Benth.,* [Longhua Hospital Affiliated to Shanghai University of Traditional Chinese Medicine], 30 g		
	Dried plant of *Oldenlandia diffusa,* [Longhua Hospital Affiliated to Shanghai University of Traditional Chinese Medicine], 30 g		
Ju et al. (2017)	**Formula I: (tonifying qi)**	N	N
	Dried roots of *Astragalus membranaceus (Fisch.)*, [Shanghai Pulmonary Hospital], 30 g		
	Dried rhizoma of *Atractylodes macrocephala Koidz*, [Shanghai Pulmonary Hospital], 15 g		
	Dried sclerotium of *Poria cocos (Schw.) Wolf*, [Shanghai Pulmonary Hospital], 15 g		
	Dried root of *Angelica sinensis (Oliv.) Diels,* [Shanghai Pulmonary Hospital], 15 g		
	Dried aerial part of *Agrimonia pilosa Ldb.*, [Shanghai Pulmonary Hospital], 30 g		
	Dried plant of *Houttuynia cordata Thunb.* [Shanghai Pulmonary Hospital], 30 g		
	Dried seed of *Benincasa hispida (Thunb.) Cogn.*, [Shanghai Pulmonary Hospital], 30 g		
	Dried seed kernel of *Coix lacryma-jobi L.var.ma-yuen (Roman.) Stapf,* [Shanghai Pulmonary Hospital], 30 g		
	Dried pericarp of *Citrus reticulate Blanco,* [Shanghai Pulmonary Hospital], 10 g		
	Dried rhizomsze of *Ligusticum chuanxiong Hort.,* [Shanghai Pulmonary Hospital], 9 g		
	Dried plant of *Salvia chinensis Benth.,* [Shanghai Pulmonary Hospital], 30 g		
	Dried plant of *Oldenlandia diffusa,* [Shanghai Pulmonary Hospital], 30 g		
	Dried root and rhizoma of *Glycyrrhiza uralensis Fisch.,* [Shanghai Pulmonary Hospital], 6 g		
	**Formula II (tonifying qi and yin)**		
	Dried roots of *Astragalus membranaceus (Fisch.)*, [Shanghai Pulmonary Hospital], 15 g		
	Dried tuberous root of *Ophiopogon japonicus (Thunb.) Ker. Gawl.,* [Shanghai Pulmonary Hospital], 15 g		
	Dried tuberous root of *Asparagus cochinchinensis(Lour.) Merr.,* [Shanghai Pulmonary Hospital], 15 g		
	Dried root of *Glehnia littoralis Fr. Schmidt ex Miq.,* [Shanghai Pulmonary Hospital], 15 g		
	Dried rhizome of *Dioscorea* opposite Thunb., [Shanghai Pulmonary Hospital], 30 g		
	Dried fruit of *Ligustrum lucidum Ait.,* [Shanghai Pulmonary Hospital], 15 g		
	Dried aerial part of *Eclipta prostrate L.,* [Shanghai Pulmonary Hospital], 15 g		
	Dried plant of *Salvia chinensis Benth.,* [Shanghai Pulmonary Hospital], 30 g		
	Dried plant of *Oldenlandia diffusa,* [Shanghai Pulmonary Hospital], 30 g		
	Fried fruit of *Setaria italica (L.) Beauv.*, [Shanghai Pulmonary Hospital], 15 g		
	Fried fruit of *Hordeurn vulgare L.*, [Shanghai Pulmonary Hospital], 15 g		
	Dried fruit of *Crataegus pinnatifida Bge.* var. *Major N.E.Br.*, [Shanghai Pulmonary Hospital], 15 g		
Chen. (2016)	**Formula I (tonifying yin)**	N	N
	Dried root of *Glehnia littoralis Fr. Schmidt ex Miq.,* [Longhua Hospital Affiliated to Shanghai University of Traditional Chinese Medicine], 15 g		
	Dried tuberous root of *Asparagus cochinchinensis(Lour.) Merr.,* [Longhua Hospital Affiliated to Shanghai University of Traditional Chinese Medicine], 15 g		
	Dried fruit of *Ligustrum lucidum Ait.,* Longhua Hospital Affiliated to Shanghai University of Traditional Chinese Medicine], 12 g		
	Dried spikes of *Prunella vulgaris L.*, [Longhua Hospital Affiliated to Shanghai University of Traditional Chinese Medicine], 15 g		
	Dried plant of *Selaginella doederleinii Hieron*, [Longhua Hospital Affiliated to Shanghai University of Traditional Chinese Medicine], 30 g		
	**Formula II (tonifying qi and yin)**		
	Dried root of Glehnia littoralis Fr. *Schmidt ex Miq*., [Longhua Hospital Affiliated to Shanghai University of Traditional Chinese Medicine], 15 g		
	Dried tuberous root of Asparagus cochinchinensis(Lour.) Merr., [Longhua Hospital Affiliated to Shanghai University of Traditional Chinese Medicine], 15 g		
	Dried fruit of Ligustrum lucidum Ait., Longhua Hospital Affiliated to Shanghai University of Traditional Chinese Medicine], 12 g		
	Dried spikes of Prunella vulgaris L., [Longhua Hospital Affiliated to Shanghai University of Traditional Chinese Medicine], 15 g		
	Dried plant of Selaginella doederleinii Hieron, [Longhua Hospital Affiliated to Shanghai University of Traditional Chinese Medicine], 30 g		
	Dried rhizoma of Astragalus membranaceus (Fisch.), [Longhua Hospital Affiliated to Shanghai University of Traditional Chinese Medicine], 30 g		
	Dried roots of Pseudostellaria heterophylla (Miq.) Pax ex Pax et Hoffm., [Longhua Hospital Affiliated to Shanghai University of Traditional Chinese Medicine], 12 g		

### Quality Assessment of Included Studies

Analysis of randomization methods showed that one study used central randomization on the internet (Jiao et al., 2019), whereas seven studies used random number table. One study did not report the randomization procedures used. Allocation concealment and blinding were not addressed in most studies. Only two studies (Jiao et al., 2019; Zhao et al., 2020) used placebo in the control group, therefore blinding of participants and personnel was not performed in most of the included studies. Only one study reported assessor blinding (Jiao et al., 2019). Two trials included in this review did not report withdrawals or dropouts and were rated “unclear” attrition risk (Feng et al., 2019; Feng et al., 2020). One study ((Ju et al., 2017) had inaccurate PFS finding whereas, one study (Shen et al., 2019) did not report ORR, and these were rated “high” reporting risk. Although all trials reported that they were RCTs, one study (Zhao et al., 2021) used the term “retrospectively” in their report, which may be an error in writing, therefore rated “unclear” in other bias.

**FIGURE 2:**
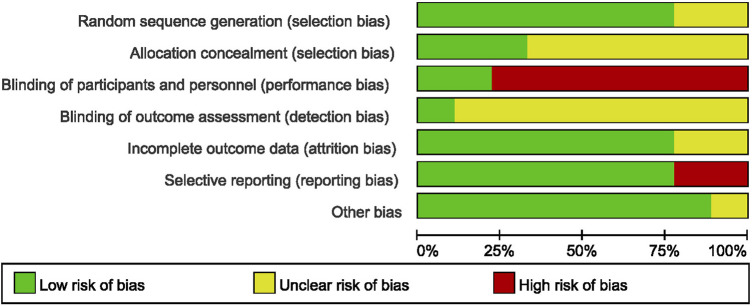
Risk of bias graph.

Risk of bias assessment of included studies are summarized in Figure 2. Risk of bias of each study was analyzed using forest maps and presented as a dot plot. A: random sequence generation (selection bias); B: allocation concealment (selection bias); C: blinding of participants and personnel (performance bias); D: blinding of outcome assessment (detection bias); E: incomplete outcome data (attrition bias); F: selective reporting (reporting bias); G: other bias.

### Primary Outcomes: Progression-Free Survival

**FIGURE 3:**
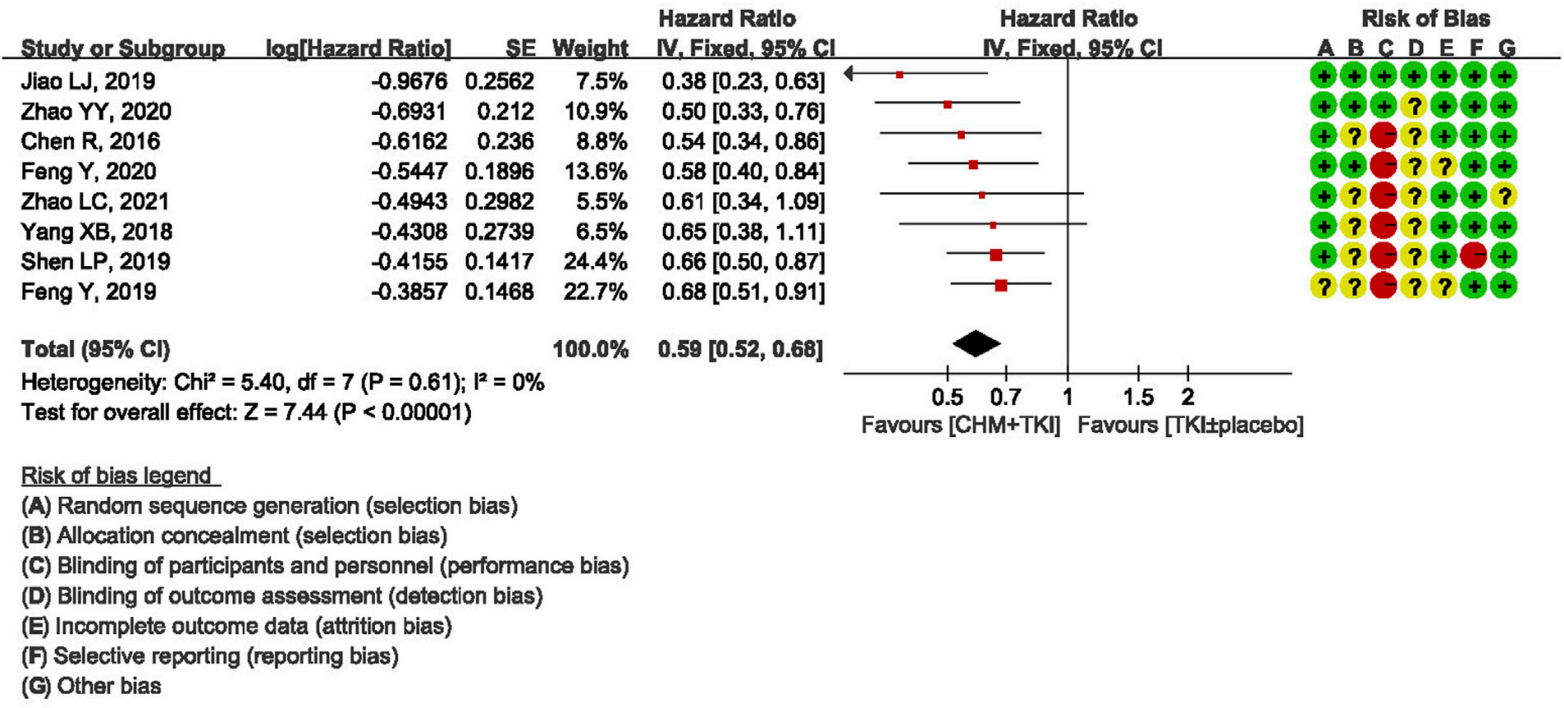
Forest plot showing PFS.

All studies reported mPFS. Notably, one study (Ju et al., 2017) reported mPFS using wrong statistical method, therefore eight studies were included in pooled analysis. Analysis of the eight studies showed a statistically significant increase in PFS in CHM + TKIs group compared with the control group (HR, 0.59; 95% CI, 0.52–0.68; *p* < 0.00001; Figure 3). No heterogeneity was observed among the studies (Chi^2^ = 5.40, df = 7; I^2^ = 0%).

### Secondary Outcomes

#### Objective Response

**FIGURE 4:**
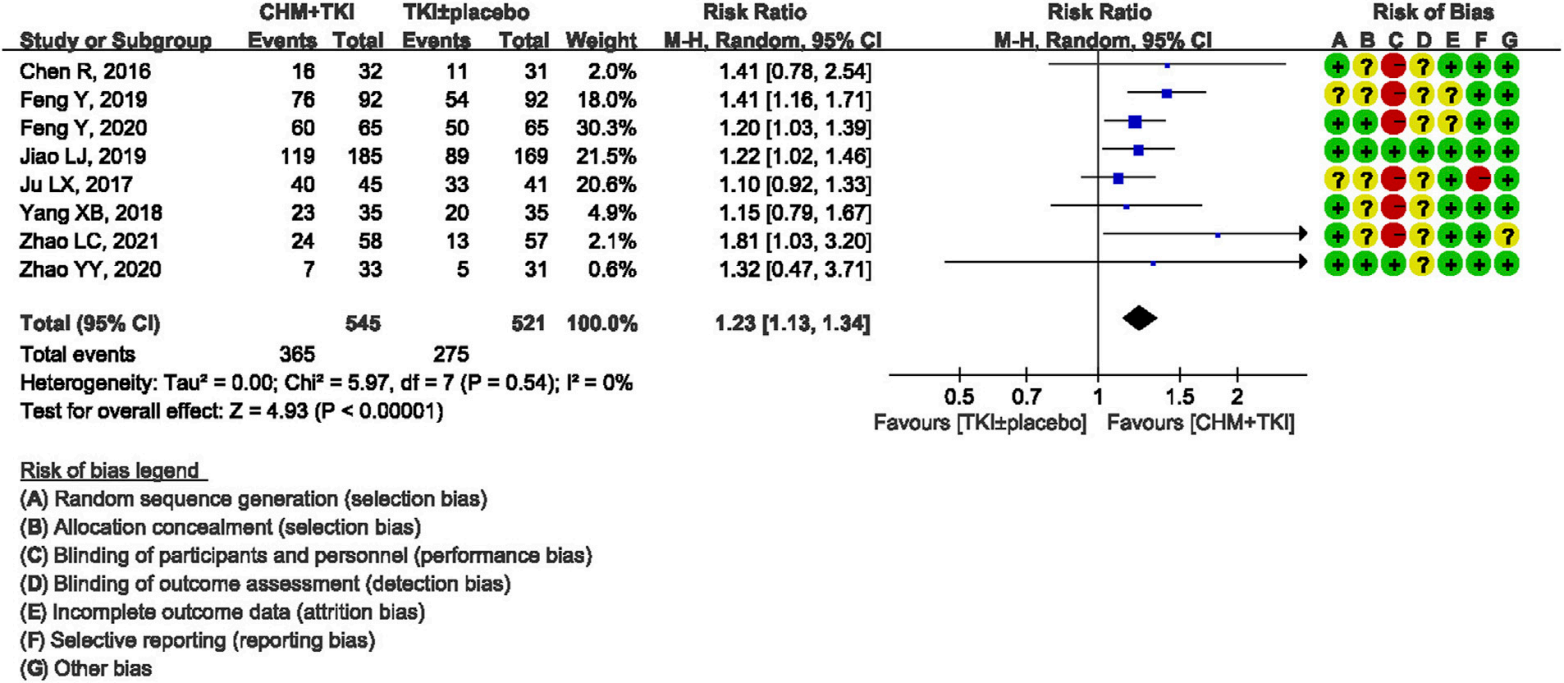
Forest plot showing ORR.

ORR was reported in eight trials involving 1,063 patients. There was no heterogeneity detected among the studies (Chi^2^ = 5.97, df = 7; I^2^ = 0%). Analysis of the pooled data showed a significantly higher ORR in TKIs + CHM group compared with TKIs ± placebo control group (RR, 1.23; 95% CI, 1.13–1.34; *p* < 0.00001; Figure 4).

In addition, DCR was reported in eight trials involving 1,063 patients. Analysis showed high heterogeneity among studies (Chi^2^ = 70.38, df = 7; I^2^ = 90%). High heterogeneity was also observed in subgroup analysis based on choice of EGFR-TKIs (gefitinib) and CHM tonifying Qi. However, subgroup analysis showed the benefit of CHM in enhancing DCR of EGFR-TKIs when applied as first line treatment (RR, 1.20; 95% CI, 1.10–1.30; *p*<0.0001) and with CHM tonifying Qi and Yin (RR, 1.20; 95% CI, 1.09–1.33; *p*=0.0004) with no or low heterogeneity (Supplementary Figures S1, S2).

#### Survival

One study (Yang et al., 2018) reported MST whereas one study (Feng et al., 2019) reported the 2-years survival rate. Therefore, median survival time and 1/2-years survival rate were not pooled.

#### Performance Status

**FIGURE 5:**
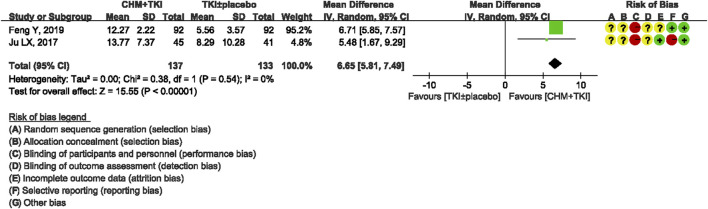
Forest plot showing changes in KPS.

A total of four studies reported performance status before and after treatment. Furthermore, two studies (Feng et al., 2019; Ju et al., 2017) including 276 patients assessed KPS before and after treatment. Meta-analysis showed significantly improved KPS in TKIs + CHM compared with the control group (Md, 6.65; 95% CI, 5.81–7.49; *p* < 0.00001) with low between-study heterogeneity (Chi^2^ = 0.38; I^2^ = 0%; Figure 5). One study (Zhao et al., 2021) reported KPS changes based on three categories (improvement, stable, and declination) and therefore was not included in the pooled data. One study (Chen et al., 2016) measured performance status using ECOG and therefore this study was not pooled.

#### Quality of Life

Two studies (Chen. 2016; Jiao et al., 2019) reported QoL using FACT-L and LCSS. Jiao et al. (2019) used categories (improvement, stable and declination) and reported improvement ratios in FACT-L 7°months postbaseline, which were significantly higher in TKIs + CHM than in TKIs + placebo group (20.54 vs 15.98%, *p* = 0.0160). For the LCSS pulmonary symptoms score, the improvement ratios in the TKIs + CHM group were significantly higher than the TKIs + placebo arm in overall QoL, overall symptomatic distress, and normal activity. (Chen et al., 2016) reported scores of FACT-L and LCSS at enrollment and 1°month postbaseline, and showed more significant improvement in TKIs + CHM than in the TKIs monotherapy regarding physical, emotional, functional well-being, Lung Cancer Subscale and all symptoms evaluated by LCSS. The two studies used different reporting methods, therefore, the results could not be pooled. One study (Shen et al., 2019) reported QLQ-C30 measurement at enrollment and 1°month postbaseline, which showed more significant improvement in TKIs + CHM than in the TKIs monotherapy group regarding physical function and overall health status.

### Adverse Events

Four studies reported incidence of overall AE. Meta-analysis showed significantly fewer total toxicities in TKIs + CHM group compared with the control group (RR, 0.68; 95% CI, 0.53–0.89; *p* = 0.004; Supplementary Figure S3) with no heterogeneity among studies (Chi^2^ = 2.11. df = 3; I^2^ = 0%).

**FIGURE 6:**
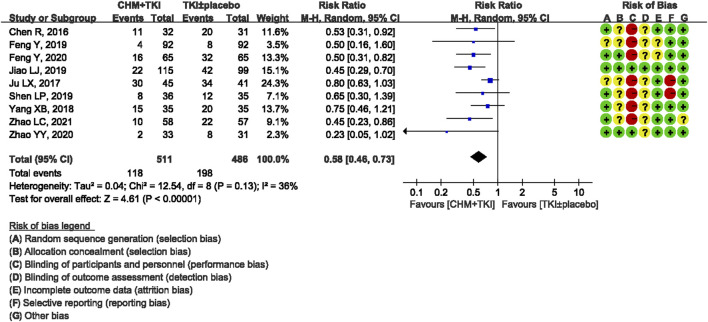
Forest plot showing incidence of cutaneous toxicity.

**FIGURE 7:**
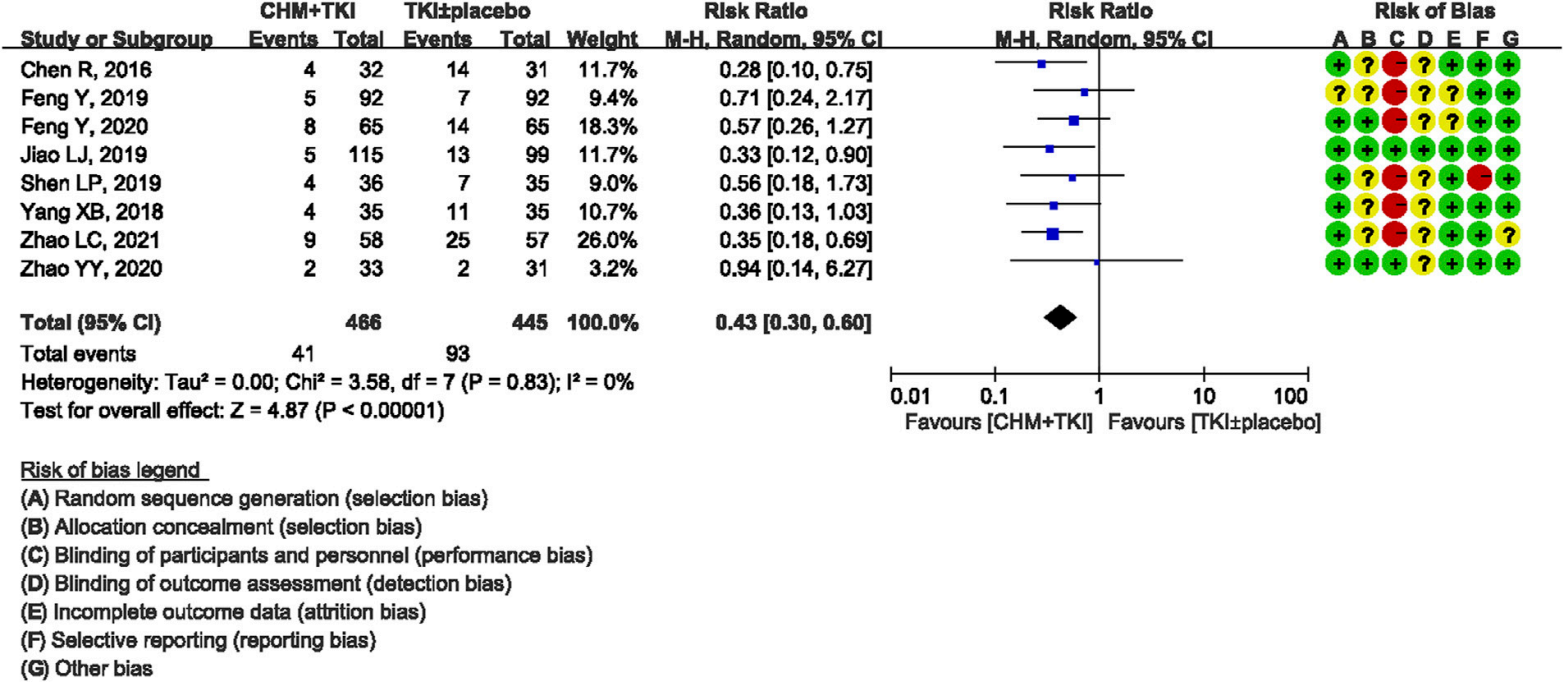
Forest plot on incidence of diarrhea.

**FIGURE 8:**
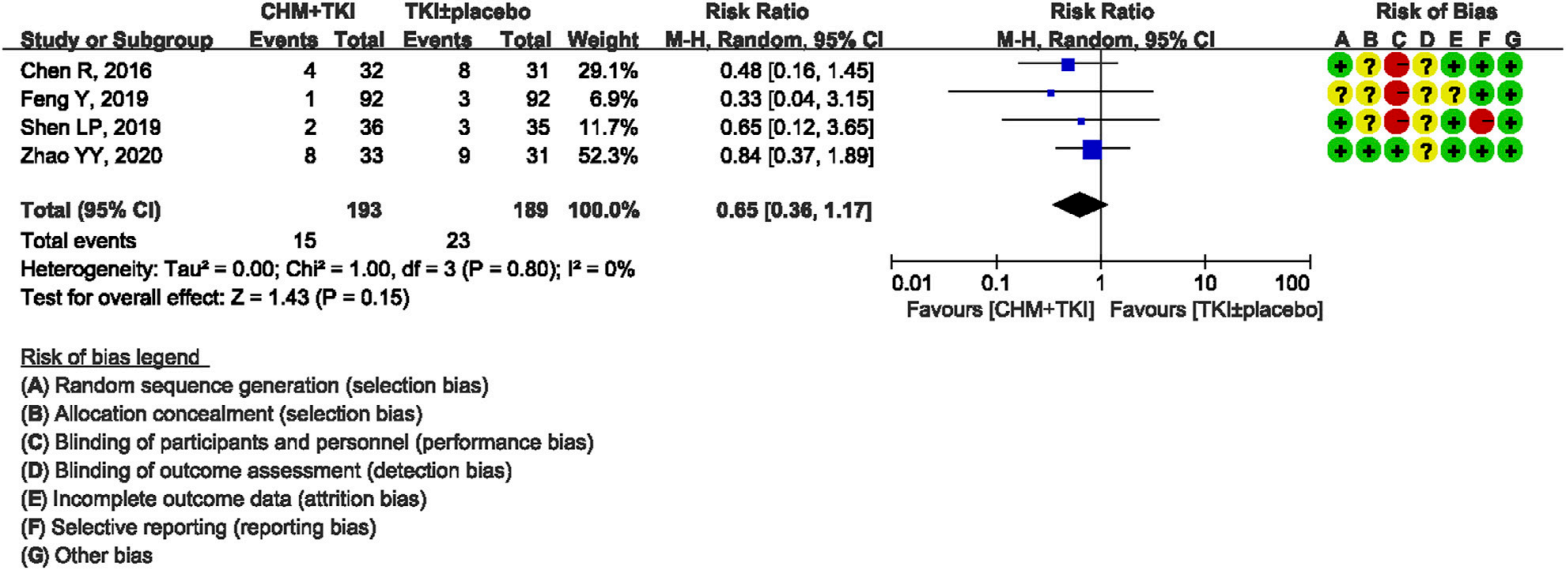
Forest plot for incidence of hepatic dysfunction.

Analysis of specific toxicity showed that all nine studies (*n* = 1,137) reported cutaneous toxicity, eight studies (*n* = 1,051) reported diarrhea, four studies (*n* = 382) reported hepatic dysfunction, and three studies (*n* = 205) reported dental ulcers. Incidence of cutaneous toxicity was significantly lower in CHM + TKI group compared with the control group (RR, 0.58; 95% CI, 0.46–0.73; *p* < 0.00001) (Figure 6) and analysis showed moderate between-study heterogeneity (Chi^2^ = 12.54, df = 8; I^2^ = 36%). Incidence of diarrhea (RR, 0.43; 95% CI, 0.30–0.60; *p* < 0.00001; Figure 7) were significantly lower in CHM + TKI group compared with the incidence in the control group, with no between-study heterogeneity. Analysis showed no significant difference in hepatic dysfunction (RR, 0.65; 95% CI, 0.36–1.17; *p* = 0.15; Figure 8) and dental ulcer (RR, 1.26; 95% CI, 0.71–2.24; *p* = 0.43) (Supplementary Figure S4) in the current study.

SAEs were few in both groups, and were reported only by Ju et al., 2017 (rash, 0 vs 14.6%), Yang et al., 2018 (diarrhea, 0 vs 5.7%; ulcer, 0 vs 2.9%), and Chen. 2016 (rash, ulcer, diarrhea and ILD, one case for each).

### Bias in Meta-Analysis

**FIGURE 9:**
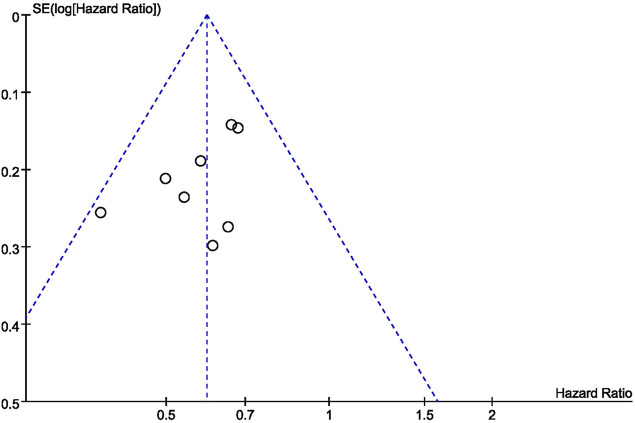
Funnel plot for PFS.

Generation of a funnel plot showed that the sample size used in this study was not large (8 studies) and estimated effect size did not vary significantly (Figure 9). Therefore, the estimated effect size points were scattered at the top of the funnel plot, and funnel plots were roughly symmetrical. Therefore, the bias of our studies was relatively small.

## Discussion

### Summary of Evidence

This study hypothesized that combination of CHM and EGFR-TKIs may prolong PFS, increase tumor response, improve performance status, and reduce toxicities in advanced NSCLC patients harboring EGFR sensitizing mutation, compared with use of EGFR-TKIs alone. A total of nine studies were included in this study.

Although no restrictions on EGFR-TKIs selection were performed in the search strategy, only three first-generation EGFR-TKIs were evaluated in the included studies. Although comparison in head-to-head clinical trials were not performed, erlotinib, gefitinib, and icotinib showed equivalent efficacy in several meta-analyses including twelve phase III RCTs involving 1,821 participants (Liang et al., 2014). Second- and third-generation EGFR-TKIs exhibit superior efficacy compared with first-generation EGFR-TKIs (Yang et al., 2015; Ramalingam et al., 2020). However, their applications were restricted by availability and health insurance policy of mainland China. Therefore, to the best of our knowledge, studies evaluating adjuvant efficacy of CHM in combination with second or third-generation EGFR-TKIs are limited.

All forms of CHM (decoction, Chinese patent medicine, pill, tablet, capsule, injection etc.) were included in the search strategy, and all the nine studies selected after filtration used oral CHM decoction as intervention method. TCM theory states that CHM should be used based on TCM syndrome, and six out of the nine studies included syndrome diagnosis criteria. Clinical experience of TCM and epidemiological investigations report that healthy Qi and/or Yin deficiency is the most common TCM syndrome in patients receiving EGFR-TKIs (Xu et al., 2017; Yu et al., 2019; Jiang et al., 2020). CHM prescriptions in the nine studies mainly aimed to tonify Qi and/or Yin (Table 1 and Table 2). Representative Qi and/or yin tonifying formula and corresponding TCM syndrome pattern were described previously by (Jiao et al., 2019).

### Comparison With Previous Studies

Several systematic reviews and meta-analyses have reported the efficacy and safety of combining CHM with EGFR-TKIs. However, these studies had several limitations. Firstly, participants with unknown EGFR status were included in almost all of systematic reviews (Zhang et al., 2018; Sui et al., 2020). EGFR-TKIs are targeted therapy for NSCLC with sensitive mutation. Notably, patients with wild EGFR gene type do not respond to EGFR-TKIs. Inclusion of patients with unknown EGFR status introduces heterogeneity into the study population and is meaningless from clinical perspective. Therefore, although our study included fewer clinical trials and participants compared with previous studies, the findings are more reliable due to the strict and proper inclusion criteria used. Secondly, PFS was not used as the primary outcome, and incomplete and improperly reported in most of the studies included, therefore most studies could not be pooled (Zhang, 2016). Up to 70–80% ORR was observed in patients with sensitizing mutations undergoing EGFR-TKI monotherapy (Mok et al., 2009; Fukuoka et al., 2011; Rosell et al., 2012; Zhou et al., 2011; Shi et al., 2013; Shi et al., 2017), therefore, PFS rather than ORR is the unmet need and first concern when combining CHM with EGFR-TKIs. Thirdly, a few studies and systematic reviews use AE of EGFR-TKIs rather than long-term efficacy as primary outcomes (Chen et al., 2019). Therefore, they had different CHM prescriptions, and were excluded in our study.

### Strengths and Limitations

The current study explored the benefit of CHM in prolonging PFS and delaying acquired resistance to EGFR-TKIs. Exon 19del and Exon 21L858R are common, but different kinds of NSCLC with different treatment responses and prognosis (Yang et al., 2015). Subgroup analysis of Jiao et al., 2019 showed a significant increase in PFS in TKIs + CHM group for patients with EGFR exon19 del, not for exon 21 L858R. However, due to the limited data, pooled analysis can’t be performed in the current study to shed light on the clinically important question.

Notably, CHM improved ORR when combined with EGFR-TKIs. With the development of new generation gene test and discovery of the mechanism of primary resistance to EGFR-TKIs, ORR of EGFR-TKIs is reported to be approximately 70–80% (Siegel et al., 2020; Fukuoka et al., 2011; Mok et al., 2009; Zhou et al., 2011; Rosell et al., 2012; Shi et al., 2017), and higher compared with ORR reported in all the nine clinical studies included in the current review. Risk of low quality in gene test may explain the relatively lower ORR and adjuvant effect of CHM in enhancing ORR.

Contrary to studies aiming to reduce adverse effects of EGFR-TKIs with CHM, all the nine studies included used PFS as primary outcome. However, side effects were reduced in most of the studies. Rash and diarrhea were effectively prevented or relieved by administration of CHM. Gefitinib, the mostly used EGFR-TKIs in China, is associated with 9.4% > Grade 3 hepatic dysfunction (Mok et al., 2009). Although CHM is associated with drug-induced hepatitis, analysis in the current study showed that incidence of hepatic dysfunction was not enhanced by adding CHM to EGFR-TKIs.

This study had a few limitations that compromise reliability of results and conclusion. Firstly, only two studies used placebo with similar taste and smell as CHM in the control group, and open label design of most of the studies introduced bias into the review. Secondly, some studies (Feng et al., 2019; Feng et al., 2020) included large number of patients in stage III resulting in superior outcome compared with studies that only included stage IV patients, which may make the overall results too optimistic. Thirdly, all studies were conducted in mainland China, therefore, there is concern on applicability of the findings outside China. Fourthly, ORR reported in all nine studies was relatively low. Primary resistance to first-generation EGFR-TKIs should be carefully detected by gene test with bigger panel before enrollment.

Further studies with multi-center, placebo-controlled, well-designed blinding method should be performed. In addition, secondary exon 20 T790M should be explored in studies using first-generation EGFR-TKIs due to its potential benefit (Mok et al., 2017). Moreover, due to the superior efficacy of third-generation EGFR-TKI compared with first-generation EGFR-TKIs (Ramalingam et al., 2020), studies should explore adjuvant effect of CHM in combination with third-generation EGFR-TKI. Furthermore, cost performance analysis should be performed for CHM when combined with EGFR-TKIs.

## Conclusion

The findings of this systematic review show that combination of CHM with EGFR-TKIs significantly delays acquired resistance and improves objective response to EGFR-TKIs. Furthermore, CHM reduces adverse effects of EGFR-TKIs. However, these findings must be interpreted with caution due to some high or unclear risk of bias of included trials.

## Data Availability

The original contributions presented in the study are included in the article/Supplementary Material, further inquiries can be directed to the corresponding authors.
