# Supplementary material for: Chinese Herbal Medicine Combined With First-Generation EGFR-TKIs in Treatment of Advanced Non-Small Cell Lung Cancer With EGFR Sensitizing Mutation: A Systematic Review and Meta-Analysis
Source: Front Pharmacol. 2021 Aug 27;12:698371. doi: 10.3389/fphar.2021.698371 (PMC8429791; doi:10.3389/fphar.2021.698371)
Supplement: Supplementary file 1 [file DataSheet3.DOCX]

Supplementary Figures

**
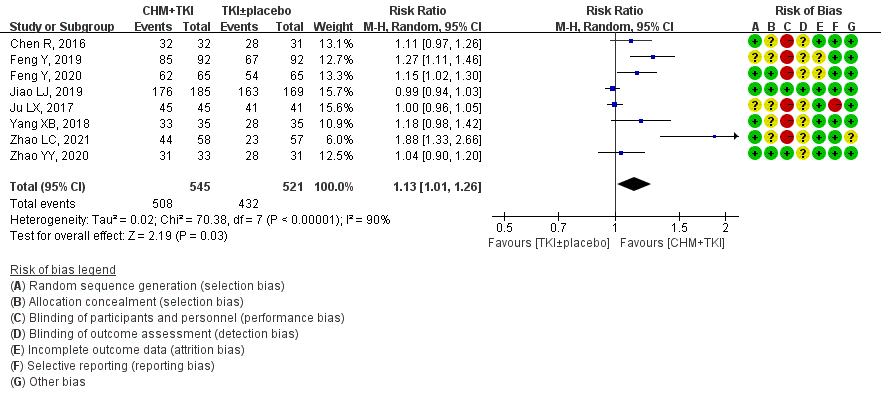
Supplementary Figure 1.** Forest Plot of DCR

**
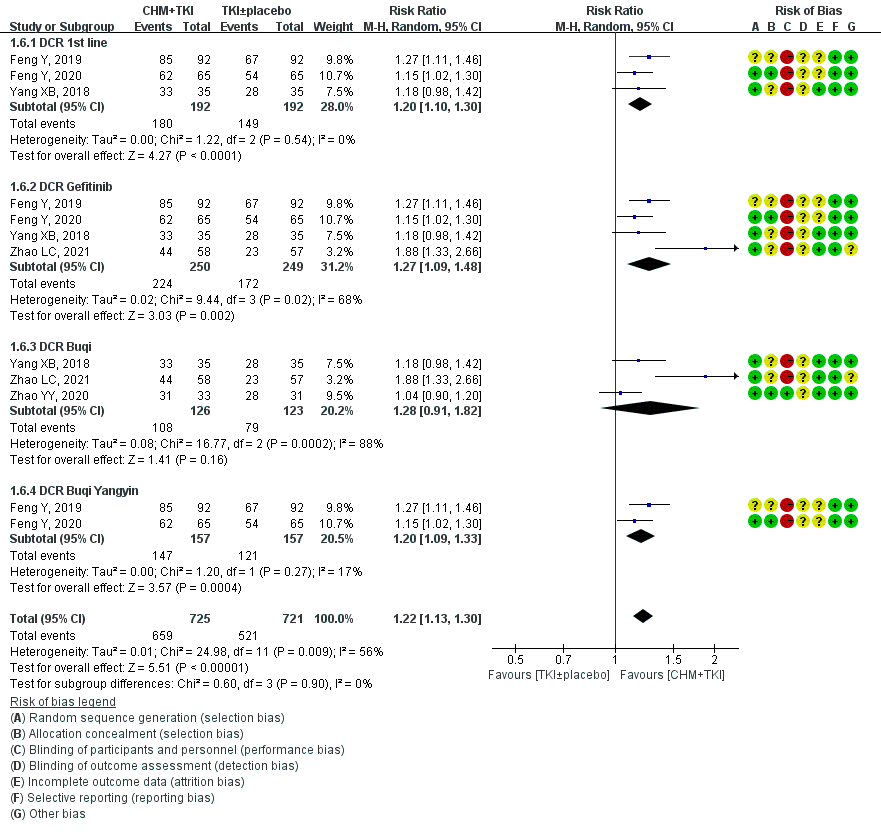
Supplementary Figure 2.** Forest Plot of Subgroup of DCR

**
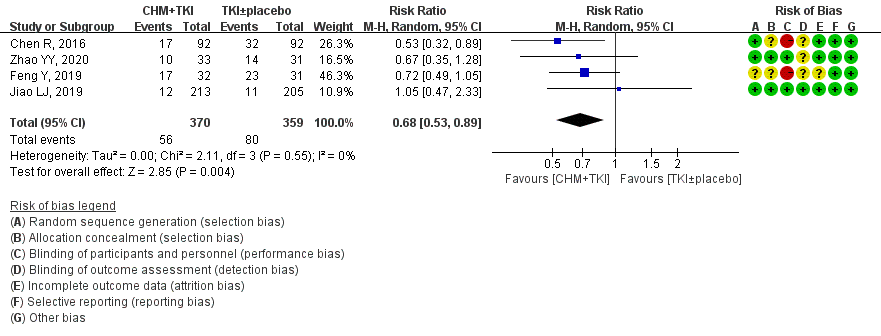
Supplementary Figure 3.** Forest Plot on incidence of AE

**
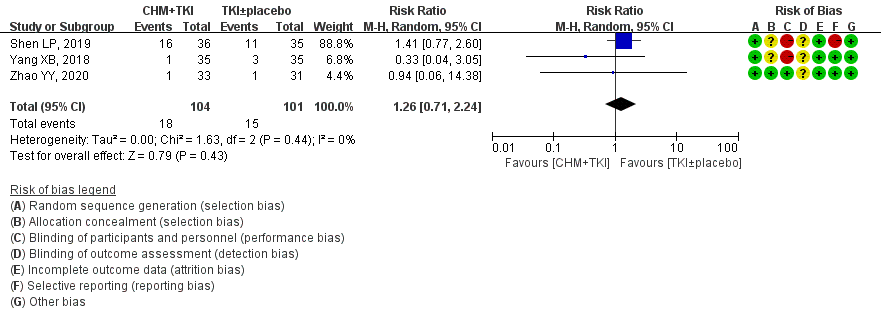
Supplementary Figure 4.** Forest Plot on incidence of dental ulcer


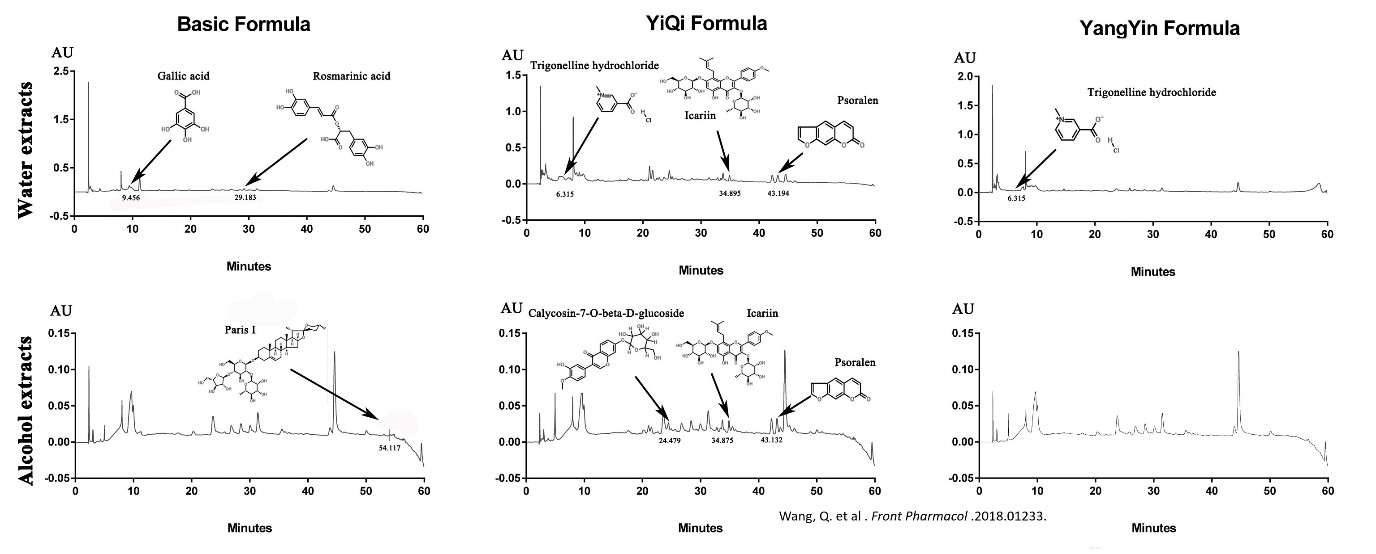


**Supplementary Figure 5.** Chemical characterisation of “Jiao LJ, 2019” Study
