# Supplementary figures and images for: Chinese Herbal Medicine Combined With First-Generation EGFR-TKIs in Treatment of Advanced Non-Small Cell Lung Cancer With EGFR Sensitizing Mutation: A Systematic Review and Meta-Analysis
Source: Front Pharmacol. 2021 Aug 27;12:698371. doi: 10.3389/fphar.2021.698371 (PMC8429791; doi:10.3389/fphar.2021.698371)

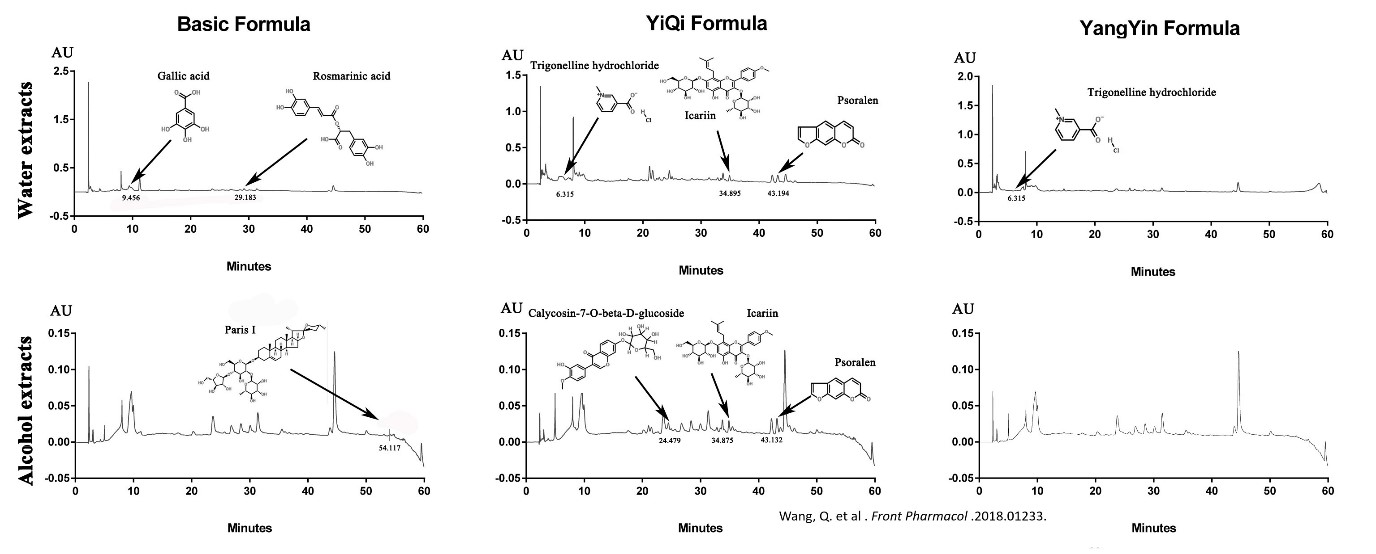

Supplement: Supplementary file 2 [file Image1.JPEG]

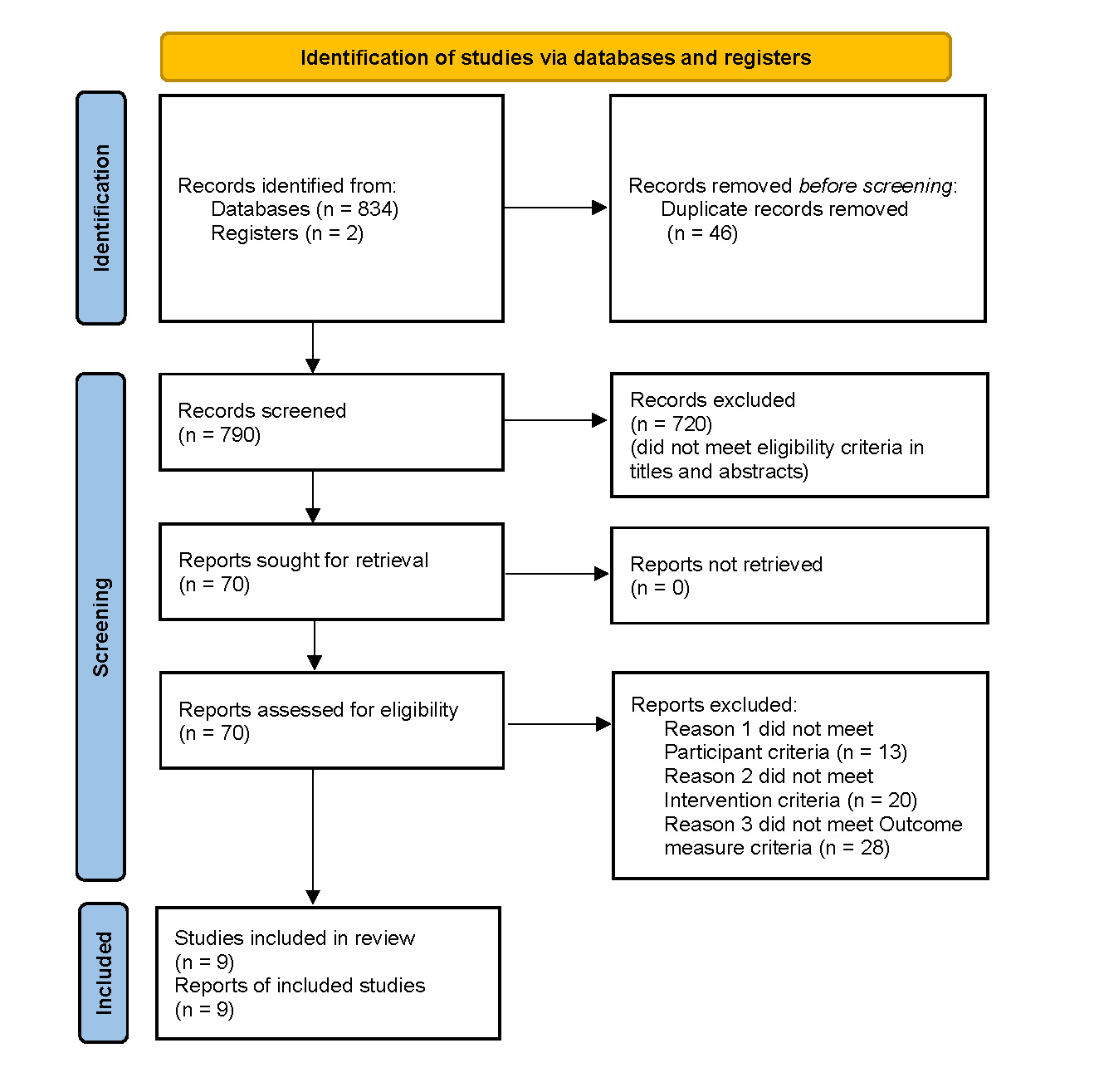

Supplement: Supplementary file 3 [file DataSheet2.ZIP › Presentation/Fig.1 PRISMA flow diagram.jpg]

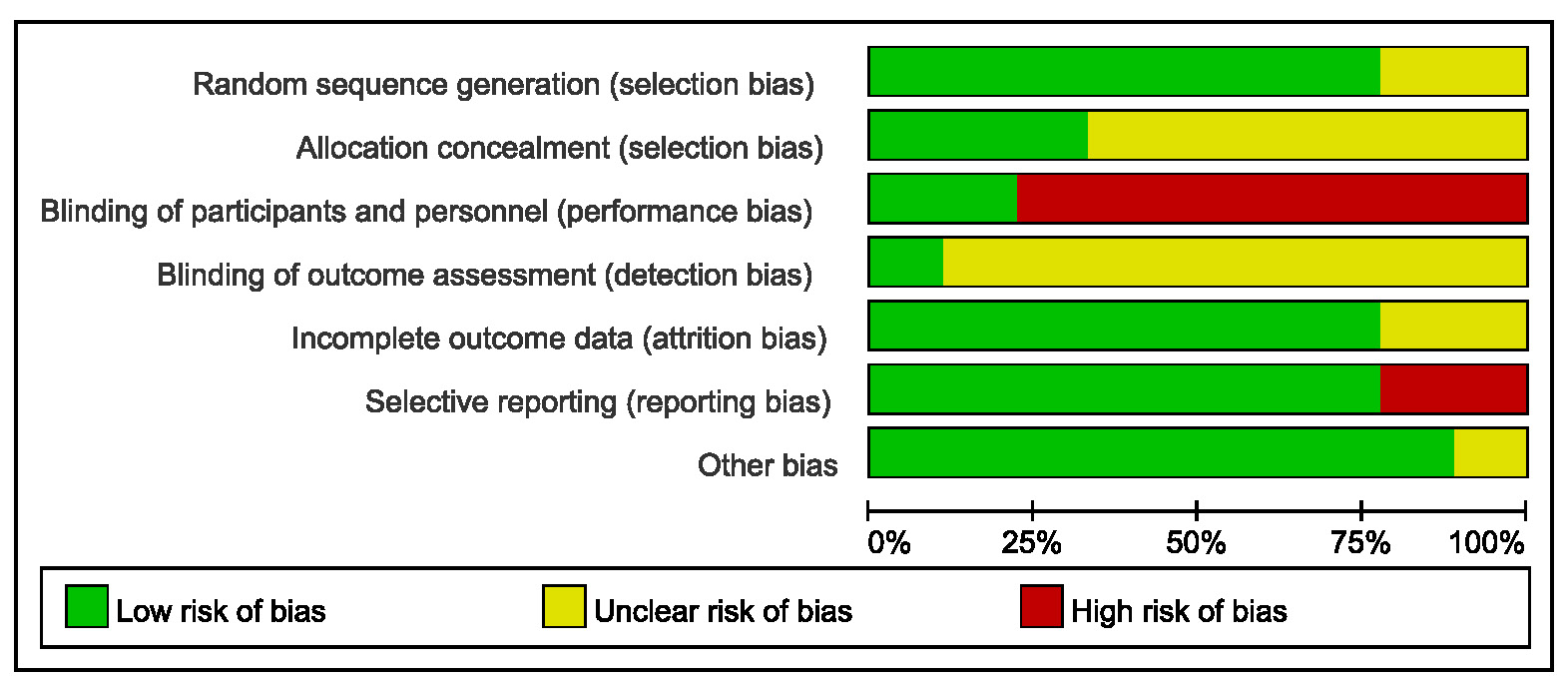

Supplement: Supplementary file 3 [file DataSheet2.ZIP › Presentation/Fig.2 Risk of bias graph.jpg]

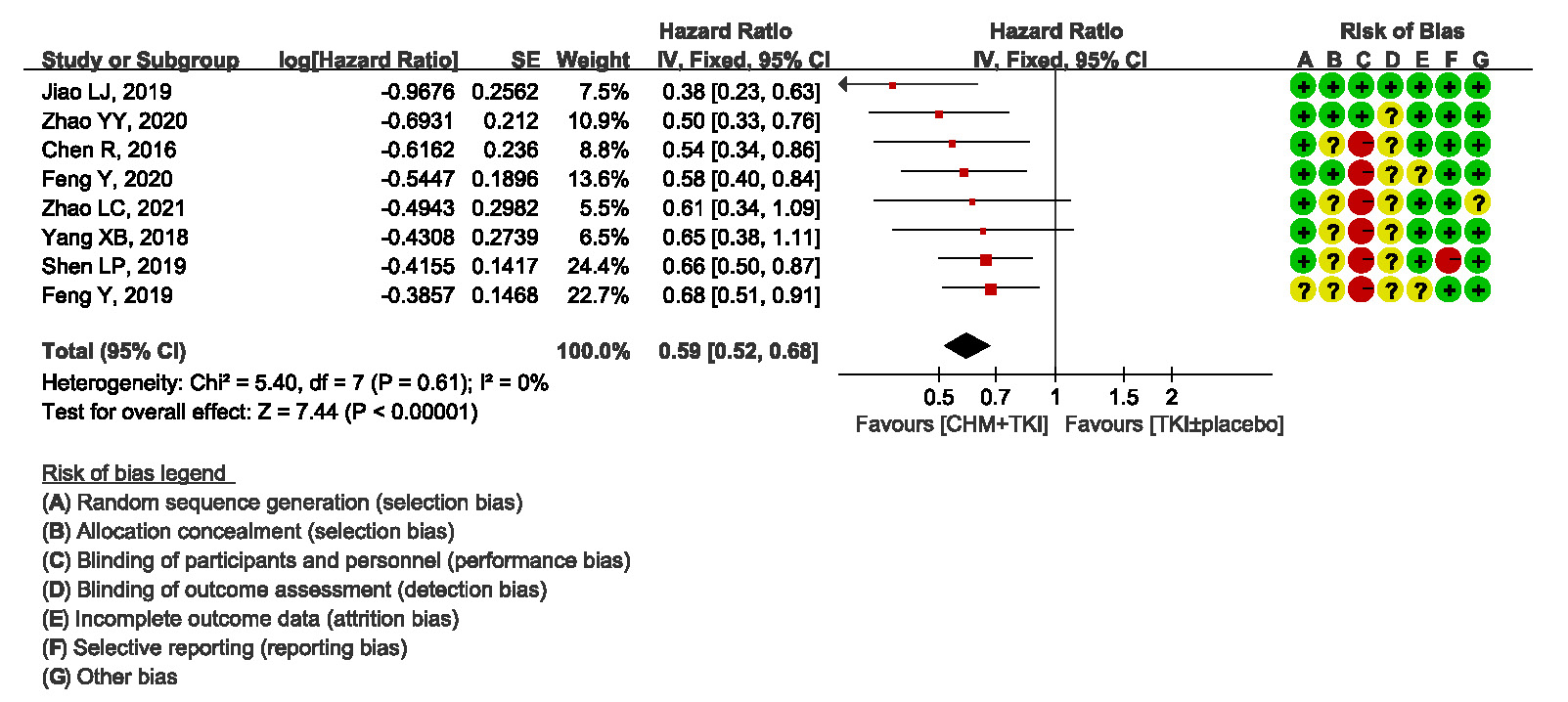

Supplement: Supplementary file 3 [file DataSheet2.ZIP › Presentation/Fig.3 Forest plot showing PFS.jpg]

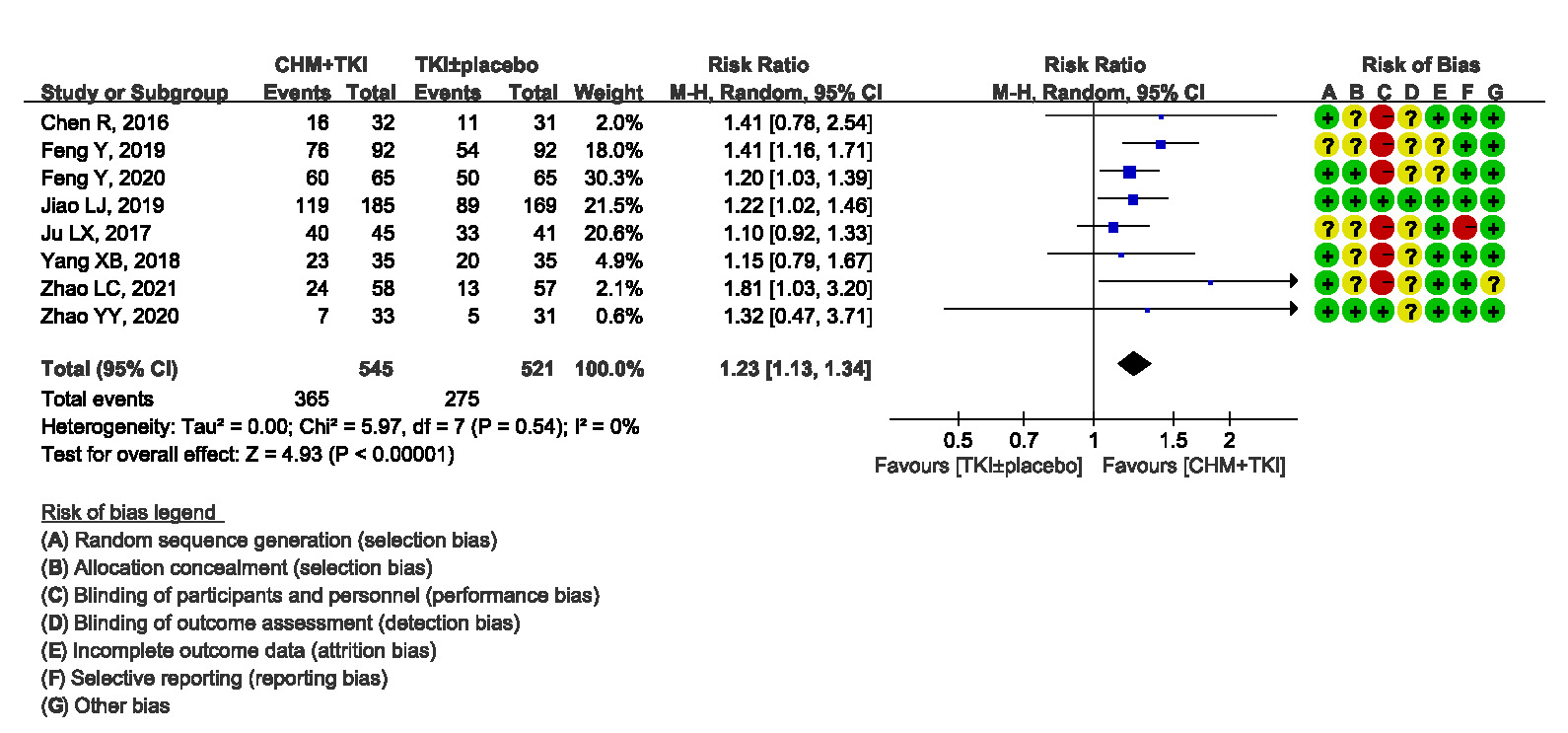

Supplement: Supplementary file 3 [file DataSheet2.ZIP › Presentation/Fig.4 Forest plot showing ORR.jpg]

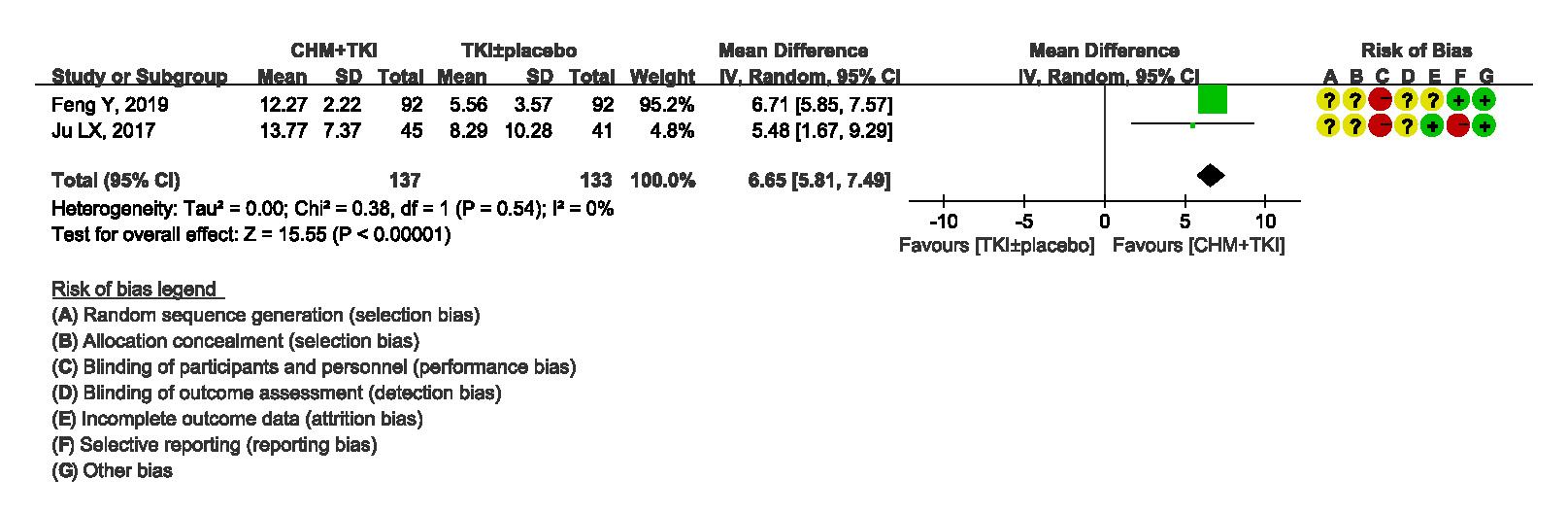

Supplement: Supplementary file 3 [file DataSheet2.ZIP › Presentation/Fig.5 Forest plot showing changes in KPS.jpg]

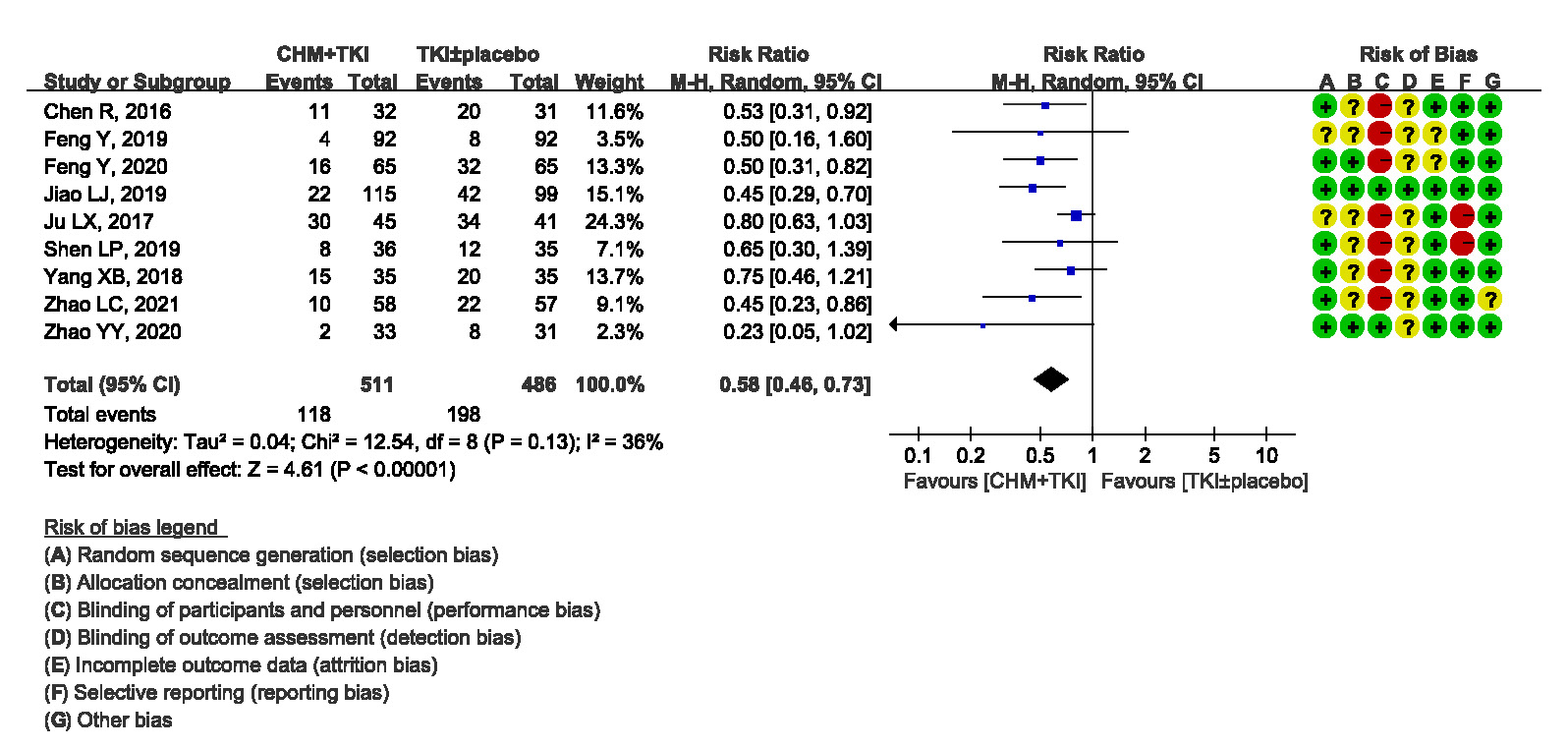

Supplement: Supplementary file 3 [file DataSheet2.ZIP › Presentation/Fig.6 Forest plot showing cutaneous toxicity.jpg]

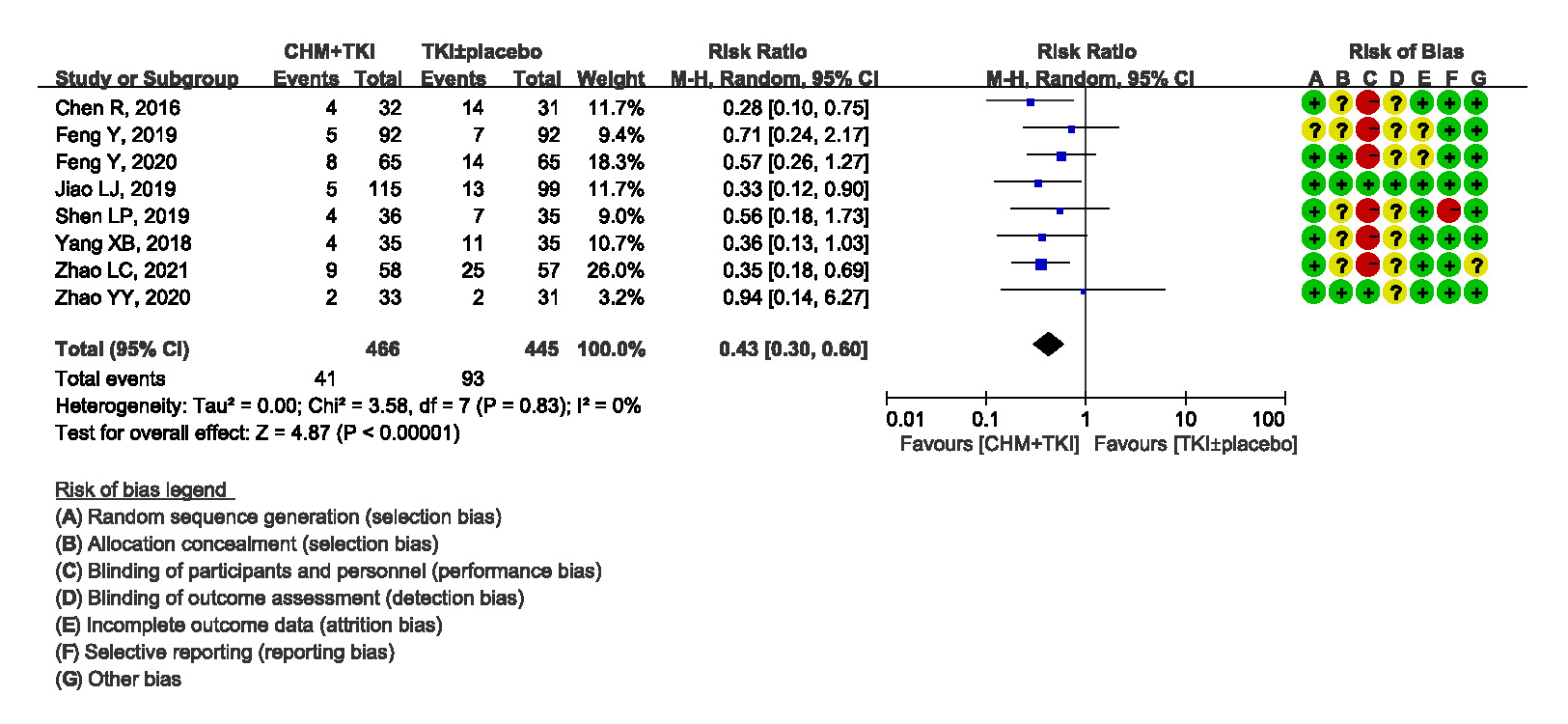

Supplement: Supplementary file 3 [file DataSheet2.ZIP › Presentation/Fig.7 Forest plot on incidence of diarrhea.jpg]

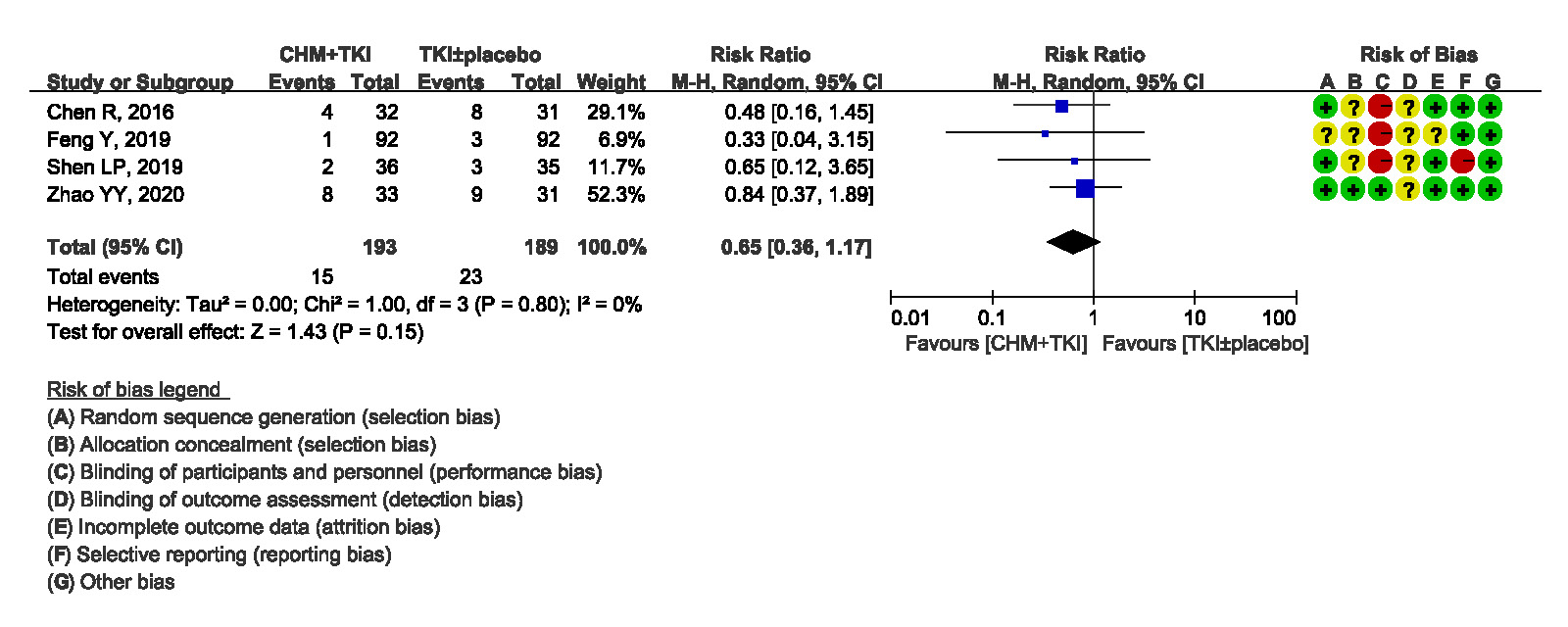

Supplement: Supplementary file 3 [file DataSheet2.ZIP › Presentation/Fig.8 Forest plot for incidence of hepatic dysfunction.jpg]

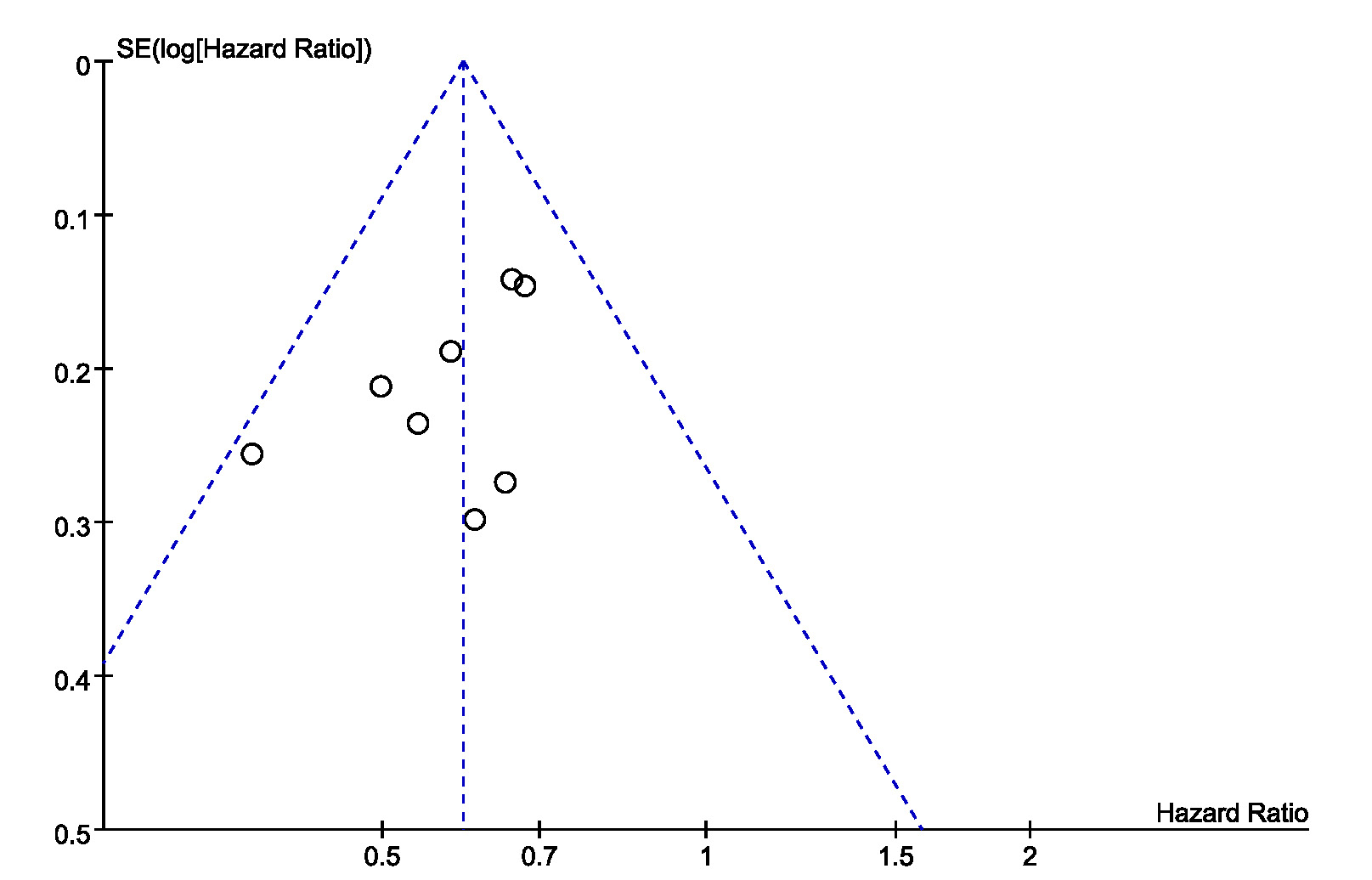

Supplement: Supplementary file 3 [file DataSheet2.ZIP › Presentation/Fig.9 Funnel plot for PFS.jpg]
