# Supplementary material for: Chinese Herbal Medicine Combined With First-Generation EGFR-TKIs in Treatment of Advanced Non-Small Cell Lung Cancer With EGFR Sensitizing Mutation: A Systematic Review and Meta-Analysis
Source: Front Pharmacol. 2021 Aug 27;12:698371. doi: 10.3389/fphar.2021.698371 (PMC8429791; doi:10.3389/fphar.2021.698371)
Supplement: Supplementary file 4 [file DataSheet1.DOCX]

(((((("prescription"[Title/Abstract] OR "recipe"[Title/Abstract]) OR "Formula"[Title/Abstract]) OR "tang"[Title/Abstract]) OR "Decoction"[Title/Abstract]) OR "injection"[Title/Abstract]) AND ("clinical trial"[Publication Type] OR "randomized controlled trial"[Publication Type])) AND (((((((((((((((("carcinoma, non-small-cell lung"[MeSH Terms] OR (((("carcinoma, non-small-cell lung"[MeSH Terms] OR (("carcinoma"[All Fields] AND "non small cell"[All Fields]) AND "lung"[All Fields])) OR "non-small-cell lung carcinoma"[All Fields]) OR (((("non"[All Fields] AND "small"[All Fields]) AND "cell"[All Fields]) AND "lung"[All Fields]) AND "cancer"[All Fields])) OR "non small cell lung cancer"[All Fields])) OR (("adenocarcinoma of lung"[MeSH Terms] OR ("adenocarcinoma"[All Fields] AND "lung"[All Fields])) OR "adenocarcinoma of lung"[All Fields])) OR ((((("epithelial cells"[MeSH Terms] OR ("epithelial"[All Fields] AND "cells"[All Fields])) OR "epithelial cells"[All Fields]) OR ("squamous"[All Fields] AND "cell"[All Fields])) OR "squamous cell"[All Fields]) AND (("lung neoplasms"[MeSH Terms] OR ("lung"[All Fields] AND "neoplasms"[All Fields])) OR "lung neoplasms"[All Fields]))) OR ((("large"[All Fields] OR "largely"[All Fields]) OR "larges"[All Fields]) AND (("cells"[MeSH Terms] OR "cells"[All Fields]) OR "cell"[All Fields]) AND (((("lung neoplasms"[MeSH Terms] OR ("lung"[All Fields] AND "neoplasms"[All Fields])) OR "lung neoplasms"[All Fields]) OR ("lung"[All Fields] AND "cancer"[All Fields])) OR "lung cancer"[All Fields]))) OR ((((("carcinoma, non-small-cell lung"[MeSH Terms] OR (("carcinoma"[All Fields] AND "non small cell"[All Fields]) AND "lung"[All Fields])) OR "non-small-cell lung carcinoma"[All Fields]) OR "nsclc"[All Fields]) OR "nsclc s"[All Fields]) OR "nsclcs"[All Fields])) OR (((("lung neoplasms"[MeSH Terms] OR ("lung"[All Fields] AND "neoplasms"[All Fields])) OR "lung neoplasms"[All Fields]) OR ("lung"[All Fields] AND "cancer"[All Fields])) OR "lung cancer"[All Fields])) OR (("lung neoplasms"[MeSH Terms] OR ("lung"[All Fields] AND "neoplasms"[All Fields])) OR "lung neoplasms"[All Fields])) OR (((("adenocarcinoma of lung"[MeSH Terms] OR ("adenocarcinoma"[All Fields] AND "lung"[All Fields])) OR "adenocarcinoma of lung"[All Fields]) OR ("lung"[All Fields] AND "adenocarcinoma"[All Fields])) OR "lung adenocarcinoma"[All Fields])) OR ((((("epithelial cells"[MeSH Terms] OR ("epithelial"[All Fields] AND "cells"[All Fields])) OR "epithelial cells"[All Fields]) OR ("squamous"[All Fields] AND "cell"[All Fields])) OR "squamous cell"[All Fields]) AND (((("lung neoplasms"[MeSH Terms] OR ("lung"[All Fields] AND "neoplasms"[All Fields])) OR "lung neoplasms"[All Fields]) OR ("lung"[All Fields] AND "cancer"[All Fields])) OR "lung cancer"[All Fields]))) OR (((("carcinoma, non-small-cell lung"[MeSH Terms] OR (("carcinoma"[All Fields] AND "non small cell"[All Fields]) AND "lung"[All Fields])) OR "non-small-cell lung carcinoma"[All Fields]) OR (((("non"[All Fields] AND "small"[All Fields]) AND "cell"[All Fields]) AND "lung"[All Fields]) AND "carcinoma"[All Fields])) OR "non small cell lung carcinoma"[All Fields])) OR ((((("epithelial cells"[MeSH Terms] OR ("epithelial"[All Fields] AND "cells"[All Fields])) OR "epithelial cells"[All Fields]) OR ("squamous"[All Fields] AND "cell"[All Fields])) OR "squamous cell"[All Fields]) AND ("lung"[MeSH Terms] OR "lung"[All Fields]) AND ((("carcinoma"[MeSH Terms] OR "carcinoma"[All Fields]) OR "carcinomas"[All Fields]) OR "carcinoma s"[All Fields]))) AND ("clinical trial"[Publication Type] OR "randomized controlled trial"[Publication Type])) AND (((((((((((((((((((((((((((("protein-tyrosine kinases"[MeSH Terms] OR ("protein tyrosine"[All Fields] AND "kinases"[All Fields])) OR "protein tyrosine kinases"[All Fields]) OR ("tyrosine"[All Fields] AND "kinase"[All Fields])) OR "tyrosine kinase"[All Fields]) AND ((((("antagonists and inhibitors"[MeSH Subheading] OR ("antagonists"[All Fields] AND "inhibitors"[All Fields])) OR "antagonists and inhibitors"[All Fields]) OR "inhibitors"[All Fields]) OR "inhibitor"[All Fields]) OR "inhibitor s"[All Fields])) OR (("gefitinib"[MeSH Terms] OR "gefitinib"[All Fields]) OR "gefitinib s"[All Fields])) OR (((("erlotinib hydrochloride"[MeSH Terms] OR ("erlotinib"[All Fields] AND "hydrochloride"[All Fields])) OR "erlotinib hydrochloride"[All Fields]) OR "erlotinib"[All Fields]) OR "erlotinib s"[All Fields])) OR ("icotinib"[Supplementary Concept] OR "icotinib"[All Fields])) OR ("afatinib"[MeSH Terms] OR "afatinib"[All Fields])) OR ("dacomitinib"[Supplementary Concept] OR "dacomitinib"[All Fields])) OR ("osimertinib"[Supplementary Concept] OR "osimertinib"[All Fields])) OR ("rociletinib"[Supplementary Concept] OR "rociletinib"[All Fields])) OR (("abivertinib"[Supplementary Concept] OR "abivertinib"[All Fields]) OR "ac0010"[All Fields])) OR (("naquotinib"[Supplementary Concept] OR "naquotinib"[All Fields]) OR "asp8273"[All Fields])) OR (("eai045"[Supplementary Concept] OR "eai045"[All Fields]) OR "eai045"[All Fields])) OR "HM61713"[All Fields]) OR "BI1482694"[All Fields]) OR (("nazartinib"[Supplementary Concept] OR "nazartinib"[All Fields]) OR "egf816"[All Fields])) OR (("osimertinib"[Supplementary Concept] OR "osimertinib"[All Fields]) OR "azd9291"[All Fields])) OR (("rociletinib"[Supplementary Concept] OR "rociletinib"[All Fields]) OR "co 1686"[All Fields])) OR (("gefitinib"[MeSH Terms] OR "gefitinib"[All Fields]) OR "zd1839"[All Fields])) OR ((("erbb receptors"[MeSH Terms] OR ("erbb"[All Fields] AND "receptors"[All Fields])) OR "erbb receptors"[All Fields]) OR "egfr"[All Fields])) OR "TKI"[All Fields]) OR ((("gefitinib"[MeSH Terms] OR "gefitinib"[All Fields]) OR "iressa"[All Fields]) OR "gefitinib s"[All Fields])) OR ((((("erlotinib hydrochloride"[MeSH Terms] OR ("erlotinib"[All Fields] AND "hydrochloride"[All Fields])) OR "erlotinib hydrochloride"[All Fields]) OR "erlotinib"[All Fields]) OR "tarceva"[All Fields]) OR "erlotinib s"[All Fields])) OR (("osimertinib"[Supplementary Concept] OR "osimertinib"[All Fields]) OR "tagrisso"[All Fields])) OR ((((((((("target"[All Fields] OR "targetability"[All Fields]) OR "targetable"[All Fields]) OR "targeted"[All Fields]) OR "targeting"[All Fields]) OR "targetings"[All Fields]) OR "targets"[All Fields]) OR "targetted"[All Fields]) OR "targetting"[All Fields]) AND ((((((("medicin"[All Fields] OR "medicinal"[All Fields]) OR "medicinally"[All Fields]) OR "medicinals"[All Fields]) OR "medicine"[MeSH Terms]) OR "medicine"[All Fields]) OR "medicine s"[All Fields]) OR "medicines"[All Fields]))) AND ("clinical trial"[Publication Type] OR "randomized controlled trial"[Publication Type]))) AND ((((((((((((((((((((((((((((((((((((((((((((((((((((((((("chin med"[Journal] OR ("chinese"[All Fields] AND "medicine"[All Fields])) OR "chinese medicine"[All Fields]) OR (((("medicine, chinese traditional"[MeSH Terms] OR (("medicine"[All Fields] AND "chinese"[All Fields]) AND "traditional"[All Fields])) OR "chinese traditional medicine"[All Fields]) OR (("traditional"[All Fields] AND "chinese"[All Fields]) AND "medicine"[All Fields])) OR "traditional chinese medicine"[All Fields])) OR ((((("asian continental ancestry group"[MeSH Terms] OR ((("asian"[All Fields] AND "continental"[All Fields]) AND "ancestry"[All Fields]) AND "group"[All Fields])) OR "asian continental ancestry group"[All Fields]) OR "chinese"[All Fields]) OR "chineses"[All Fields]) AND (("herbal medicine"[MeSH Terms] OR ("herbal"[All Fields] AND "medicine"[All Fields])) OR "herbal medicine"[All Fields]))) OR ((((("asian continental ancestry group"[MeSH Terms] OR ((("asian"[All Fields] AND "continental"[All Fields]) AND "ancestry"[All Fields]) AND "group"[All Fields])) OR "asian continental ancestry group"[All Fields]) OR "chinese"[All Fields]) OR "chineses"[All Fields]) AND ((((("herbal medicine"[MeSH Terms] OR ("herbal"[All Fields] AND "medicine"[All Fields])) OR "herbal medicine"[All Fields]) OR "herbalism"[All Fields]) OR "herbal"[All Fields]) OR "herbals"[All Fields]) AND "drug"[All Fields])) OR ((((("tradition"[All Fields] OR "tradition s"[All Fields]) OR "traditional"[All Fields]) OR "traditionals"[All Fields]) OR "traditions"[All Fields]) AND (("herbal medicine"[MeSH Terms] OR ("herbal"[All Fields] AND "medicine"[All Fields])) OR "herbal medicine"[All Fields]))) OR (("herbal medicine"[MeSH Terms] OR ("herbal"[All Fields] AND "medicine"[All Fields])) OR "herbal medicine"[All Fields])) OR ((((("tradition"[All Fields] OR "tradition s"[All Fields]) OR "traditional"[All Fields]) OR "traditionals"[All Fields]) OR "traditions"[All Fields]) AND (((("asian continental ancestry group"[MeSH Terms] OR ((("asian"[All Fields] AND "continental"[All Fields]) AND "ancestry"[All Fields]) AND "group"[All Fields])) OR "asian continental ancestry group"[All Fields]) OR "japanese"[All Fields]) OR "japaneses"[All Fields]) AND ((((((("medicin"[All Fields] OR "medicinal"[All Fields]) OR "medicinally"[All Fields]) OR "medicinals"[All Fields]) OR "medicine"[MeSH Terms]) OR "medicine"[All Fields]) OR "medicine s"[All Fields]) OR "medicines"[All Fields]))) OR ((("medicine, traditional"[MeSH Terms] OR ("medicine"[All Fields] AND "traditional"[All Fields])) OR "traditional medicine"[All Fields]) OR ("traditional"[All Fields] AND "medicine"[All Fields]))) OR (((((("ethnomedicinal"[All Fields] OR "ethnomedicinally"[All Fields]) OR "ethnomedicines"[All Fields]) OR "medicine, traditional"[MeSH Terms]) OR ("medicine"[All Fields] AND "traditional"[All Fields])) OR "traditional medicine"[All Fields]) OR "ethnomedicine"[All Fields])) OR (((("medicine, traditional"[MeSH Terms] OR ("medicine"[All Fields] AND "traditional"[All Fields])) OR "traditional medicine"[All Fields]) OR ("folk"[All Fields] AND "medicine"[All Fields])) OR "folk medicine"[All Fields])) OR (((("medicine, traditional"[MeSH Terms] OR ("medicine"[All Fields] AND "traditional"[All Fields])) OR "traditional medicine"[All Fields]) OR ("folk"[All Fields] AND "remedies"[All Fields])) OR "folk remedies"[All Fields])) OR (((("medicine, traditional"[MeSH Terms] OR ("medicine"[All Fields] AND "traditional"[All Fields])) OR "traditional medicine"[All Fields]) OR ("home"[All Fields] AND "remedies"[All Fields])) OR "home remedies"[All Fields])) OR (((("medicine, traditional"[MeSH Terms] OR ("medicine"[All Fields] AND "traditional"[All Fields])) OR "traditional medicine"[All Fields]) OR ("indigenous"[All Fields] AND "medicine"[All Fields])) OR "indigenous medicine"[All Fields])) OR (((("medicine, traditional"[MeSH Terms] OR ("medicine"[All Fields] AND "traditional"[All Fields])) OR "traditional medicine"[All Fields]) OR ("primitive"[All Fields] AND "medicine"[All Fields])) OR "primitive medicine"[All Fields])) OR (("materia medica"[MeSH Terms] OR ("materia"[All Fields] AND "medica"[All Fields])) OR "materia medica"[All Fields])) OR (((((((("homoeopathic remedies"[All Fields] OR "materia medica"[MeSH Terms]) OR ("materia"[All Fields] AND "medica"[All Fields])) OR "materia medica"[All Fields]) OR ("homeopathic"[All Fields] AND "remedies"[All Fields])) OR "homeopathic remedies"[All Fields]) OR "homeopathy"[MeSH Terms]) OR "homeopathy"[All Fields]) OR ("homeopathic"[All Fields] AND "remedies"[All Fields]))) OR (((("materia medica"[MeSH Terms] OR ("materia"[All Fields] AND "medica"[All Fields])) OR "materia medica"[All Fields]) OR "nosode"[All Fields]) OR "nosodes"[All Fields])) OR (((("medicine, east asian traditional"[MeSH Terms] OR ((("medicine"[All Fields] AND "east"[All Fields]) AND "asian"[All Fields]) AND "traditional"[All Fields])) OR "east asian traditional medicine"[All Fields]) OR ((("traditional"[All Fields] AND "east"[All Fields]) AND "asian"[All Fields]) AND "medicine"[All Fields])) OR "traditional east asian medicine"[All Fields])) OR ((("medicine, east asian traditional"[MeSH Terms] OR ((("medicine"[All Fields] AND "east"[All Fields]) AND "asian"[All Fields]) AND "traditional"[All Fields])) OR "east asian traditional medicine"[All Fields]) OR ((("traditional"[All Fields] AND "far"[All Fields]) AND "eastern"[All Fields]) AND "medicine"[All Fields]))) OR (((("medicine, east asian traditional"[MeSH Terms] OR ((("medicine"[All Fields] AND "east"[All Fields]) AND "asian"[All Fields]) AND "traditional"[All Fields])) OR "east asian traditional medicine"[All Fields]) OR (("far"[All Fields] AND "east"[All Fields]) AND "medicine"[All Fields])) OR "far east medicine"[All Fields])) OR (((("medicine, east asian traditional"[MeSH Terms] OR ((("medicine"[All Fields] AND "east"[All Fields]) AND "asian"[All Fields]) AND "traditional"[All Fields])) OR "east asian traditional medicine"[All Fields]) OR ("oriental"[All Fields] AND "medicine"[All Fields])) OR "oriental medicine"[All Fields])) OR (("choson uihak"[Journal] OR ("korean"[All Fields] AND "medicine"[All Fields])) OR "korean medicine"[All Fields])) OR (((("medicine, tibetan traditional"[MeSH Terms] OR (("medicine"[All Fields] AND "tibetan"[All Fields]) AND "traditional"[All Fields])) OR "tibetan traditional medicine"[All Fields]) OR ("tibetan"[All Fields] AND "medicine"[All Fields])) OR "tibetan medicine"[All Fields])) OR "herb*"[All Fields]) OR ("herbaceous"[All Fields] AND ("agent"[All Fields] OR "agents"[All Fields]))) OR (((("plants, medicinal"[MeSH Terms] OR ("plants"[All Fields] AND "medicinal"[All Fields])) OR "medicinal plants"[All Fields]) OR ("medicinal"[All Fields] AND "plant"[All Fields])) OR "medicinal plant"[All Fields])) OR (((("plants, medicinal"[MeSH Terms] OR ("plants"[All Fields] AND "medicinal"[All Fields])) OR "medicinal plants"[All Fields]) OR ("medicinal"[All Fields] AND "herbs"[All Fields])) OR "medicinal herbs"[All Fields])) OR ((((("plants, medicinal"[MeSH Terms] OR ("plants"[All Fields] AND "medicinal"[All Fields])) OR "medicinal plants"[All Fields]) OR ("medicinal"[All Fields] AND "plant"[All Fields])) OR "medicinal plant"[All Fields]) AND (((((((((((("economics"[MeSH Terms] OR "economics"[All Fields]) OR "production"[All Fields]) OR "productions"[All Fields]) OR "efficiency"[MeSH Terms]) OR "efficiency"[All Fields]) OR "productivity"[All Fields]) OR "product"[All Fields]) OR "product s"[All Fields]) OR "productive"[All Fields]) OR "productively"[All Fields]) OR "productivities"[All Fields]) OR "products"[All Fields]))) OR (((("plant extracts"[MeSH Terms] OR ("plant"[All Fields] AND "extracts"[All Fields])) OR "plant extracts"[All Fields]) OR ("plant"[All Fields] AND "extract"[All Fields])) OR "plant extract"[All Fields])) OR (((("plant preparations"[MeSH Terms] OR ("plant"[All Fields] AND "preparations"[All Fields])) OR "plant preparations"[All Fields]) OR ("plant"[All Fields] AND "preparation"[All Fields])) OR "plant preparation"[All Fields])) OR (((("plant preparations"[MeSH Terms] OR ("plant"[All Fields] AND "preparations"[All Fields])) OR "plant preparations"[All Fields]) OR ("herbal"[All Fields] AND "preparation"[All Fields])) OR "herbal preparation"[All Fields])) OR (((("botanic"[All Fields] OR "botanical"[All Fields]) OR "botanically"[All Fields]) OR "botanicals"[All Fields]) OR "botanics"[All Fields])) OR ("botany"[MeSH Terms] OR "botany"[All Fields])) OR (((("kampos"[All Fields] OR "medicine, kampo"[MeSH Terms]) OR ("medicine"[All Fields] AND "kampo"[All Fields])) OR "kampo medicine"[All Fields]) OR "kampo"[All Fields])) OR ((("medicine, kampo"[MeSH Terms] OR ("medicine"[All Fields] AND "kampo"[All Fields])) OR "kampo medicine"[All Fields]) OR "kanpo"[All Fields])) OR ((((("tradition"[All Fields] OR "tradition s"[All Fields]) OR "traditional"[All Fields]) OR "traditionals"[All Fields]) OR "traditions"[All Fields]) AND (((("medicine, mongolian traditional"[MeSH Terms] OR (("medicine"[All Fields] AND "mongolian"[All Fields]) AND "traditional"[All Fields])) OR "mongolian traditional medicine"[All Fields]) OR ("mongolian"[All Fields] AND "medicine"[All Fields])) OR "mongolian medicine"[All Fields]))) OR (((("medicine, mongolian traditional"[MeSH Terms] OR (("medicine"[All Fields] AND "mongolian"[All Fields]) AND "traditional"[All Fields])) OR "mongolian traditional medicine"[All Fields]) OR (("mongolian"[All Fields] AND "folk"[All Fields]) AND "medicine"[All Fields])) OR "mongolian folk medicine"[All Fields])) OR (((("medicine, mongolian traditional"[MeSH Terms] OR (("medicine"[All Fields] AND "mongolian"[All Fields]) AND "traditional"[All Fields])) OR "mongolian traditional medicine"[All Fields]) OR ("mongolian"[All Fields] AND "medicine"[All Fields])) OR "mongolian medicine"[All Fields])) OR (("phytotherapy"[MeSH Terms] OR "phytotherapy"[All Fields]) OR "phytotherapies"[All Fields])) OR ((("phytotherapy"[MeSH Terms] OR "phytotherapy"[All Fields]) OR ("herb"[All Fields] AND "therapy"[All Fields])) OR "herb therapy"[All Fields])) OR ((("phytotherapy"[MeSH Terms] OR "phytotherapy"[All Fields]) OR ("herbal"[All Fields] AND "therapy"[All Fields])) OR "herbal therapy"[All Fields])) OR ("ethnopharmacology"[MeSH Terms] OR "ethnopharmacology"[All Fields])) OR ("ethnobotany"[MeSH Terms] OR "ethnobotany"[All Fields])) OR ("phytogenic"[All Fields] OR "phytogenics"[All Fields])) OR (((("complementary therapies"[MeSH Terms] OR ("complementary"[All Fields] AND "therapies"[All Fields])) OR "complementary therapies"[All Fields]) OR ("alternative"[All Fields] AND "medicine"[All Fields])) OR "alternative medicine"[All Fields])) OR (((("complementary therapies"[MeSH Terms] OR ("complementary"[All Fields] AND "therapies"[All Fields])) OR "complementary therapies"[All Fields]) OR ("alternative"[All Fields] AND "therapy"[All Fields])) OR "alternative therapy"[All Fields])) OR (((("complementary therapies"[MeSH Terms] OR ("complementary"[All Fields] AND "therapies"[All Fields])) OR "complementary therapies"[All Fields]) OR ("complementary"[All Fields] AND "therapy"[All Fields])) OR "complementary therapy"[All Fields])) OR (((("complementary therapies"[MeSH Terms] OR ("complementary"[All Fields] AND "therapies"[All Fields])) OR "complementary therapies"[All Fields]) OR ("complementary"[All Fields] AND "medicine"[All Fields])) OR "complementary medicine"[All Fields])) OR (("trends cardiovasc med"[Journal] OR "case manager"[Journal]) OR "tcm"[All Fields])) OR "CHM"[All Fields]) OR ((zhong, yi[Author] OR yi, zhong[Author]) OR zhong, yi[Investigator] OR zhong yi[Author] OR zhong yi[Investigator])) OR (((((("food, formulated"[MeSH Terms] OR ("food"[All Fields] AND "formulated"[All Fields])) OR "formulated food"[All Fields]) OR "Formula"[All Fields]) OR "formula s"[All Fields]) OR "formulae"[All Fields]) OR "formulas"[All Fields])) OR "tang"[All Fields]) OR ((((("decoct"[All Fields] OR "decocted"[All Fields]) OR "decocting"[All Fields]) OR "Decoction"[All Fields]) OR "decoctions"[All Fields]) OR "decocts"[All Fields])) OR ((((((((((((((("inject"[All Fields] OR "injectability"[All Fields]) OR "injectant"[All Fields]) OR "injectants"[All Fields]) OR "injectate"[All Fields]) OR "injectates"[All Fields]) OR "injected"[All Fields]) OR "injectible"[All Fields]) OR "injectibles"[All Fields]) OR "injecting"[All Fields]) OR "injections"[MeSH Terms]) OR "injections"[All Fields]) OR "injectable"[All Fields]) OR "injectables"[All Fields]) OR "injection"[All Fields]) OR "injects"[All Fields])) AND ("clinical trial"[Publication Type] OR "randomized controlled trial"[Publication Type]))) AND (((((((((("clinical trial"[Publication Type] OR "clinical trials as topic"[MeSH Terms]) OR "clinical trial"[All Fields]) OR ((("randomized controlled trial"[Publication Type] OR "randomized controlled trials as topic"[MeSH Terms]) OR "randomized controlled trial"[All Fields]) OR "randomised controlled trial"[All Fields])) OR (("controlled clinical trial"[Publication Type] OR "controlled clinical trials as topic"[MeSH Terms]) OR "controlled clinical trial"[All Fields])) OR ((("multicenter study"[Publication Type] OR "multicenter studies as topic"[MeSH Terms]) OR "multicenter study"[All Fields]) OR "multicentre study"[All Fields])) OR (("clinical trial, phase i"[Publication Type] OR "clinical trials, phase i as topic"[MeSH Terms]) OR "phase 1 clinical trial"[All Fields])) OR (("clinical trial, phase ii"[Publication Type] OR "clinical trials, phase ii as topic"[MeSH Terms]) OR "phase 2 clinical trial"[All Fields])) OR (("clinical trial, phase iii"[Publication Type] OR "clinical trials, phase iii as topic"[MeSH Terms]) OR "phase 3 clinical trial"[All Fields])) OR (("clinical trial, phase iv"[Publication Type] OR "clinical trials, phase iv as topic"[MeSH Terms]) OR "phase 4 clinical trial"[All Fields])) AND ("clinical trial"[Publication Type] OR "randomized controlled trial"[Publication Type]))) AND ("clinical trial"[Publication Type] OR "randomized controlled trial"[Publication Type]))
